# Hydrogel and scalp/skin conductivities impact dose from tumor treating fields

**DOI:** 10.3389/fbioe.2025.1484317

**Published:** 2025-02-24

**Authors:** Edwin Lok, Olivia Liang, Monika Haack, Eric T. Wong

**Affiliations:** ^1^ Brain Tumor Center & Neuro-Oncology Unit, Beth Israel Deaconess Medical Center, Boston, MA, United States; ^2^ Division of Hematology/Oncology, Departments of Medicine, Brown University Health & Rhode Island Hospital, Providence, RI, United States; ^3^ Department of Neurology, Beth Israel Deaconess Medical Center, Boston, MA, United States; ^4^ Departments of Neurology, Medicine, Neurosurgery & Radiation Oncology, Brown University Health & Rhode Island Hospital, Providence, RI, United States

**Keywords:** hydrogel, TTFields, cancer, toxicity, scalp, skin

## Abstract

**Purpose:**

Tumor Treating Fields (TTFields) are delivered by transducer arrays applied to scalp or body surface for treatment of multiple malignancies. Dermatologic complications are thought to be related to hydrogel situated between the electrodes and scalp or skin to facilitate electric field penetration. High intensity of TTFields on these surfaces may also be a contributing factor. We explored conductivity changes in the hydrogel and skin to improve TTFields coverage and penetration.

**Methods:**

Magnetic resonance imaging datasets from 12 glioblastoma patients and attenuation-corrected positron emission tomography–computed tomography datasets from 3 non-small cell lung and 2 ovarian carcinoma patients were used to segment anatomic structures. Finite element mesh models were generated and solved for distribution of applied electric fields, rate of energy deposition, and current density at the gross tumor volume (GTV) and clinical target volume (CTV). Electric field-volume, specific absorption rate–volume, and current density-volume histograms were generated, by which plan quality metrics were used to evaluate relative differences in field coverage between models at various hydrogel and skin conductivities.

**Results:**

By varying conductivity of hydrogel, TTFields coverage at GTV or CTV increased up to 0.5 S/m for head and 1.0 S/m for thorax and pelvis models, and no additional increase was observed beyond these saturation points. Although scalp hotspots increased or decreased by +1.5%, −0.1%, and −0.9% in E_5%_, SAR_5%_, and CD_5%_, the skin hotspots increased by as much as +23.5%, +45.7%, and +20.6%, respectively. When altering conductivity of the entire scalp, TTFields coverage peaked near 1 S/m at the GTV or CTV for the head models. TTFields coverage in both the GTV and scalp increased up to 1 S/m for the head models but plateaued thereafter. Contouring under the scalp increased scalp hotspots by +316% in E_5%_ at 1 S/m compared to altering the conductivity of the entire scalp. GTV hotspots decreased by +17% in E_5%_ at 1 S/m.

**Conclusion:**

TTFields delivery can be modulated by the conductivity of hydrogel and scalp/skin at the transducer-scalp or transducer-skin interface. Optimizing this aspect of TTFields delivery may increase tumor control while minimizing toxicity at the scalp or skin.

## Introduction

Tumor Treating Fields (TTFields) therapy is the standard-of-care for patients with newly-diagnosed and recurrent glioblastomas (Stupp et al., 2017; Stupp et al., 2012). Its anti-cancer efficacy is derived from alternating electric fields tuned to a frequency of 200 kHz, which disrupt tumor cells in mitosis, cause double strand DNA breaks, and impose other types of cellular stress response resulting in secondary autophagy, immunogenic cell death or both (Gera et al., 2015; Karanam et al., 2017; Shteingauz et al., 2018; Voloshin et al., 2020). TTFields are delivered continuously to the patient’s head via two pairs of orthogonally positioned transducer arrays (Swanson et al., 2016). Daily compliance at ≥75% or continuous use for ≥18 h is an important determinant of treatment efficacy (Toms et al., 2019). Furthermore, TTFields are approved for the treatment of mesothelioma and 4 arrays are placed on the patient’s chest wall (Ceresoli et al., 2019). Randomized clinical trials also showed improved efficacy in patients with advanced staged non-small cell lung cancer when combined with immune checkpoint inhibitor (Leal et al., 2023), and those with 1–10 brain metastases when integrated with radiosurgery (Mehta et al., 2024). Therefore, TTFields will likely be incorporated as an adjuvant treatment for other malignancies in the future.

A major complication associated with continuous use of TTFields is localized breakdown of scalp or skin (Lacouture et al., 2020; Lacouture et al., 2014). These dermatologic complications, which include erythema, pruritis, and secondary infection (Lacouture et al., 2020), are thought to be a result of allergic reactions to hydrogel, which is situated between the electrodes and scalp or skin to facilitate electric field penetration, rather than from TTFields themselves emanating from the electrodes. High ambient temperature and hyperhidrosis are also modifying factors that may alter the severity of these complications (Lacouture et al., 2020). However, we postulated that the dermatologic complications are unrelated to hydrogel allergy but associated with the intensity of TTFields, and therefore we explored the possibility of optimizing the delivery of TTFields by minimizing field intensity on the scalp while maximizing it within the tumor. First, we analyzed 12 patient models (7 head models from glioblastoma patients, 3 thorax models from non-small cell carcinoma patients, and 2 pelvis models from ovarian cancer patients) and investigated the TTFields’ coverage effects on scalp/skin, skull and target(s) under different hydrogel electric conductivities. We found that there is a general difference in the optimum conductivity between head and body (thorax and pelvis) models; but variability also exists among individual patients within each anatomic treatment site. Second, to mitigate scalp irritation, we performed simulation studies by varying hydrogel and whole scalp conductivities, with the latter mimicking the effect of applying topical agents on the scalp. Changes in TTFields coverage in the scalp, skull, and target volumes were systematically explored using 17 patient models (12 glioblastoma, 3 non-small cell carcinoma, and 2 ovarian cancer patients). Finally, alterations in TTFields penetration were also investigated by varying the electric conductivity only in the region of the skin layer directly beneath the hydrogels, and we noted improved TTFields penetration into the brain when gadolinium and other non-reactive, highly conductive mixtures were injected into the scalp layer.

## Materials and methods

Magnetic resonance imaging (MRI) data from 12 glioblastoma patients, who underwent TTFields therapy, as well as positron emission tomography-computed tomography (PET-CT) imaging data from 3 lung carcinoma and 2 ovarian carcinoma patients, were used to segment intracranial, intrathoracic, and intrapelvic anatomic structures under an institutional review board approved protocol at the Dana Farber Cancer Institute. T1 post-gadolinium MR sequences were used as previously described to delineate gross tumor volumes in the brain tumor models (Lok et al., 2017). PET data were used to define the FDG-positive gross tumor volume (GTV) in the lung and pelvic models, where a uniform 3 mm expansion around the GTV was conducted to create the clinical target volume (CTV), as previously described (Lok et al., 2023; Lok et al., 2021). A 3-dimensional finite element mesh was then generated for each model in ScanIP (Synopsys, Mountain View, CA) and then imported into COMSOL Multiphysics 5.5 (COMSOL, Burlington, MA) for finite element analysis. Sensitivity analysis as a function of hydrogel electric conductivity was achieved by varying the electric conductivity parameter in log unit intervals between 0.001 and 1000 S/m for each model. The nominal value for hydrogel electric conductivity was 0.1 S/m as previously used across multiple modeling studies (Lok et al., 2017; Timmons et al., 2017; Wenger et al., 2015). Additionally, the nominal value used in this study for the scalp and skin conductivity was 0.00105 S/m. Head models were developed with hydrogel electric conductivity up to 10 S/m due to a lower propensity of electric field saturation, compared to thorax and pelvis models that had conductivity values beyond 10 S/m and up to 1000 S/m due to their greater axial diameters for electric field penetration. The electric conductivity of the whole scalp in the head was modeled from 0.001 to 10 S/m, while the electric conductivity of the skin on the body was modeled from 0.001 to 100 S/m. Furthermore, conductive fluids in the regions within the scalp or skin directly underneath each hydrogel layer were modeled in log unit increments from 0.001 to 100 S/m.

Appropriate material properties and boundary conditions were specified as previously described, and magnetic fields were assumed to be negligible at the frequency range for the operation of TTFields (Lok et al., 2017; Lok et al., 2021). The AC/DC module from COMSOL Multiphysics was used to solve for the electric field distribution in each model. To quantify the distributions as a function of hydrogel, skin, scalp, and conductive fluids, plan quality metrics (PQMs) derived from each model’s respective volume histograms, were used to make comparisons within Excel 2019 (Microsoft, Redmond, WA). PQMs included 95% coverage metrics such as E_95%_, SAR_95%_, and CD_95%_, which are electric fields strength, specific absorption rate (or the rate at which energy is absorbed), and current density received by 95% volume of a particular tissue, respectively. Median coverage for electric field, SAR and current density were also utilized to quantitatively compare between models, denoted by E_50%_, SAR_50%_, and CD_50%_, respectively. The 5% metrics such as E_5%_, SAR_5%_, CD_5%_ were used to quantify hotspots, specifically the intensity of each metric within 5% of tissue.

## Results

### TTFields distribution metrics saturate according to hydrogel electric conductivity

**FIGURE 1:**
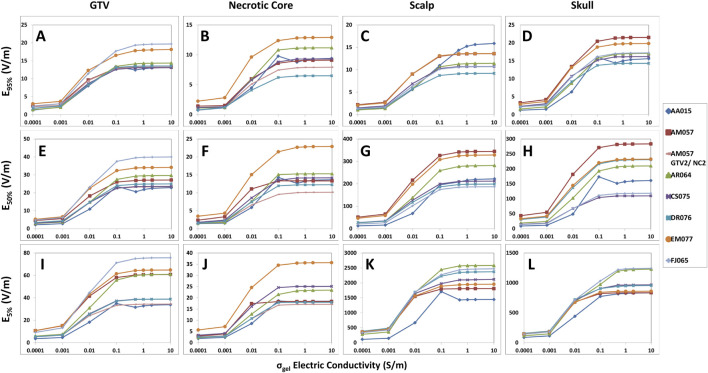
Differences in the hydrogel conductivity saturation characteristics among GTV, necrotic core, scalp and skull of the head models represented by E_95%_, E_50%_, and E_5%_ coverage metrics. When increasing hydrogel electric conductivity from 0.0001 to 10.0 S/m, E_95%_ **(A–D)**, E_50%_ **(E–H)**, and E_5%_ **(I–L)** metrics increased and saturated at 0.5 S/m. No significant increase was noted thereafter.

Our study revealed that there are important saturation points at which increasing the hydrogel electric conductivity beyond a certain value resulted in no further increase in TTFields coverage at the GTV or CTV. This saturation point appears to be different between the head and body models. For the head models, the saturation point is near 0.5 S/m and increasing the hydrogel conductivity from the nominal value of 0.1–0.5 S/m resulted an average increase of +3.4% in E_95%_, +3.6% in SAR_95%_, and +4.1% in CD_95%_ at the GTV (Figure 1A; Supplementary Figures 1A, 2A). There was negligible change in 95% TTFields coverage beyond 0.5 S/m. Similarly, the median coverage at the GTV increased on average by +2.0% in E_50%_, +5.2% in SAR_50%_, and +2.4% in CD_50%_ (Figure 1E; Supplementary Figures 1E, 2E). The GTV hotspots, denoted by E_5%_, SAR_5%_, and CD_5%_, increased by an average of +2.8%, +6.2%, and +3.6%, respectively (Figure 1I; Supplementary Figures 1I, 2I). Similar saturation characteristics were noted for necrotic core (Figure 1; Supplementary Figures 1, 2).

Within the scalp, the median coverage, represented by E_50%_, SAR_50%_, and CD_50%_, increased on average by +5.8%, +14.0%, and +6.0%, respectively (Figure 1G; Supplementary Figures 1G, 2G). The 95% coverage to the scalp followed a similar pattern of increase in intensity as a function of hydrogel electric conductivity and exhibited saturation points near 0.5 S/m (Figure 1C; Supplementary Figures 1C, 2C). However, there was negligible change in the hotspots (5%) coverage of TTFields beyond 0.5 S/m. Similar saturation characteristics were noted for skull (Figure 1; Supplementary Figures 1, 2).

**FIGURE 2:**
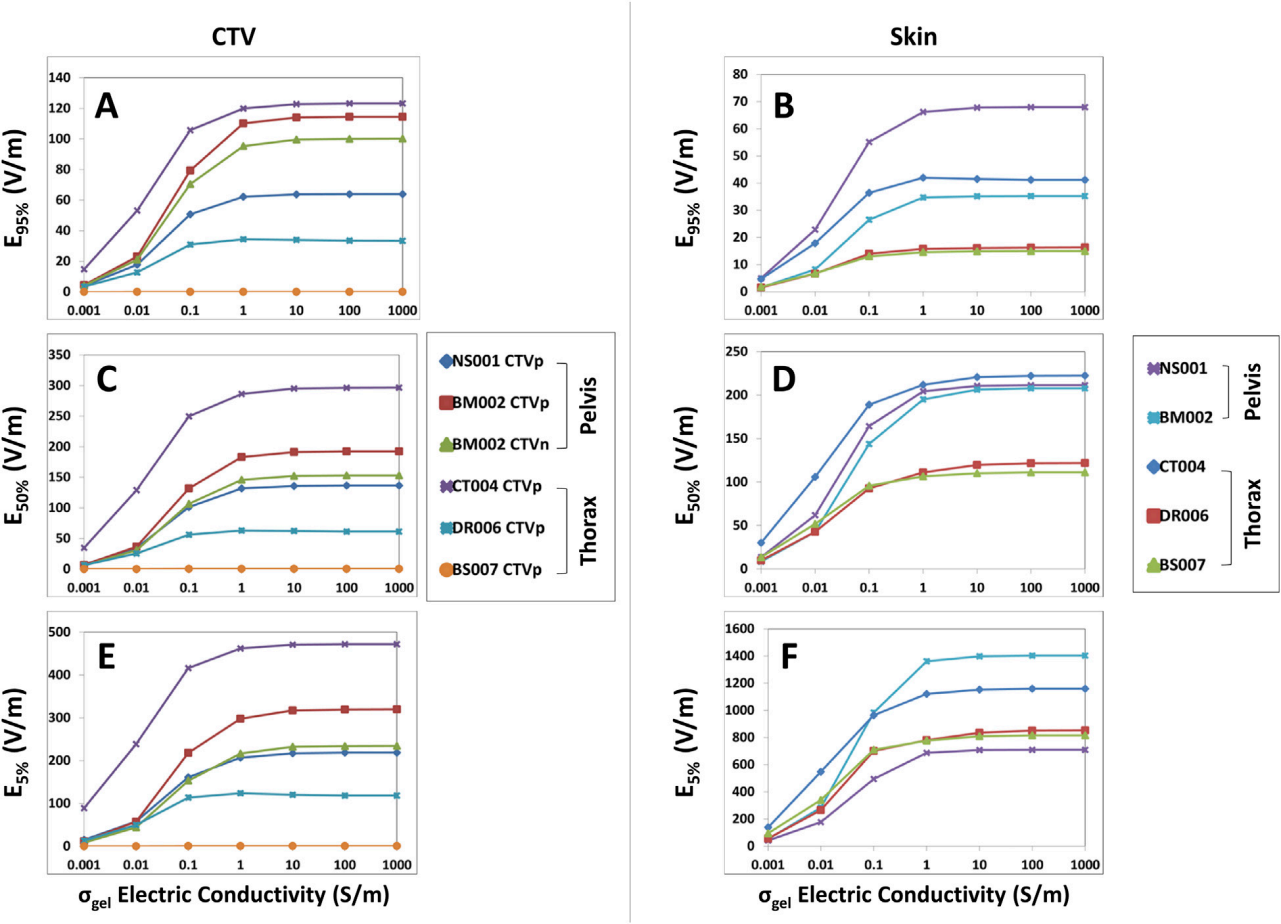
E_95%_, E_50%_, and E_5%_ coverage metrics for CTV and skin of the thoracic and pelvic models with respect to increasing hydrogel conductivity. When increasing hydrogel electric conductivity from 0.001 to 1,000.0 S/m, E_95%_ **(A, B)**, E_50%_ **(C, D)**, and E_5%_ **(E, F)** metrics increased and reached saturation points of 1.0 S/m. No significant increase was noted thereafter.

For the thorax and pelvis models, a different saturation point for hydrogel conductivity was observed near 1.0 S/m and increasing the hydrogel conductivity from 0.1 to 1.0 S/m yielded an average increase of +20.5% in E_95%_, +47.7% in SAR_95%_, and +43.1% in CD_95%_ at the CTV (Figure 2A; Supplementary Figures 3A, 4A). There was negligible change in 95% TTFields coverage beyond 1.0 S/m. The average increase in median coverage at the CTV was +22.5% for E_50%_, +50.4% for SAR_50%_, and +24.4% for CD_50%_ (Figure 2C; Supplementary Figures 3C, 4C). The CTV hotspots, denoted by E_5%,_ SAR_5%,_ and CD_5%_, increased by an average of +26.6%, +51.0%, and +21.7%, respectively (Figure 2E; Supplementary Figures 3E, 4E). In addition, the skin hotspots, denoted by E_5%_, SAR_5%_, and CD_5%_, increased by +22.8%, +45.7%, and +20.6%, respectively (Figure 2F; Supplementary Figures 3F, 4F). The 95% and median (50%) coverage to the skin also followed suit (Figures 2B, D; Supplementary Figures 3B, D, 4B, D). Collectively, these data indicate that the conductivity saturation points for the thorax and pelvis models are higher by an order of magnitude compared to the head models, and this difference has potential implications for the penetration of TTFields.

### TTFields distribution metrics peak in targets with respect to whole scalp/skin electric conductivity

**FIGURE 3:**
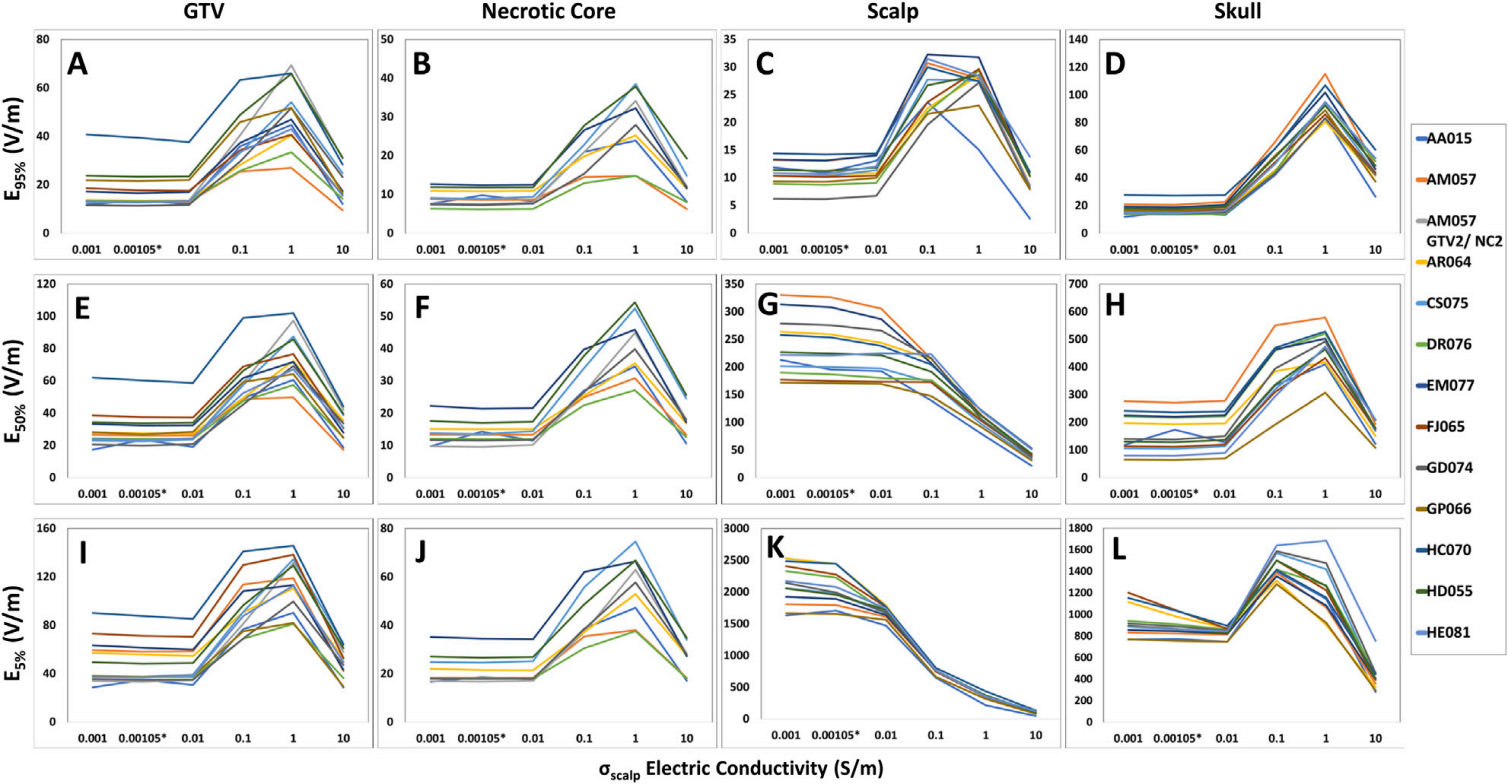
Differences in E_95%_, E_50%_, and E_5%_ coverage metrics among GTV, necrotic core, scalp and skull of the head models with respect to skin conductivity alterations. For the GTV, necrotic core, and skull, the E_95%_ **(A, B, D)**, E_50%_ **(E, F, H)**, and E_5%_ **(I, J, L)** metrics increased as the scalp conductivity increased from 0.001 to 1.0 S/m. However, a decrease was noted beyond 1.0 S/m. Comparatively, the E_95%_ **(C)** for the scalp followed the same pattern, whereas the E_50%_ **(G)** and E_5%_ **(K)** metrics decreased entirely when the scalp conductivity increased from 0.001 to 10.0 S/m.

When altering the scalp/skin electric conductivity, the TTFields coverage, SAR and current density peaked at around 1.0 S/m and then decreased after 1.0 S/m. Specifically, in the head models (n = 12), the average increase in TTFields coverage at the GTVs was +212.3%, +168.7% and +150.7% for E_95%_, E_50%_, and E_5%_, respectively (Figures 3A, E, I). Additionally, the average increase for the SAR_95%_, SAR_50%_, and SAR_5%_ were +1,005.9%, +737.9%, and +589.4%, respectively (Supplementary Figures 5A, E, I). Lastly, the average increase for the CD_95%_, CD_50%_, and CD_5%_ were +177.1%, +169.4%, and +150.7%, respectively (Supplementary Figures 6A, E, I). Similar characteristics were noted in necrotic core and skull, except for scalp where all electric field metrics other than for E_95%_ decreased with increasing conductivity and both SAR and current density increased progressively (Figure 3; Supplementary Figures 5, 6).

**FIGURE 4:**
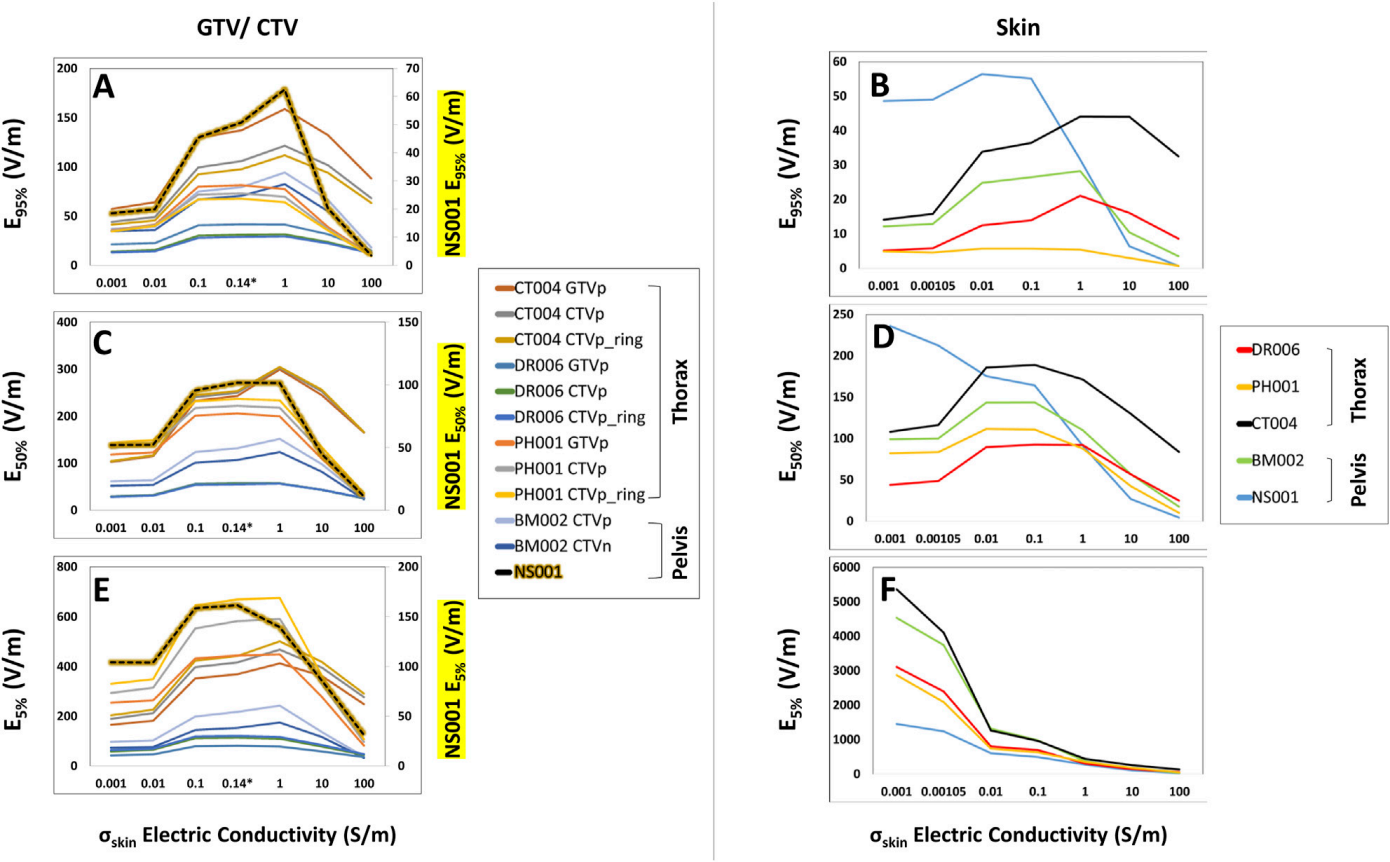
E_95%_, E_50%_, and E_5%_ coverage metrics of the GTV/CTV and skin of the body models with respect to increasing skin conductivity. As the skin conductivity increased from 0.001 to 1 S/m, the GTV/CTV and skin E_95%_, E_50%_, and E_5%_ coverage metrics **(A–E)** increased until 1.0 S/m and then decreased thereafter, with the exception of the skin E_5%_ metric that decreased with increasing conductivity **(F)**. It is also notable that the CTV from model NS001 had distinct E_95%_, E_50%_, and E_5%_ coverages, of which its values are displayed on the secondary axis.

Compared to the head models, the average increase for the thoracic GTVs (n = 3) were much lower at +3.5%, +6.6%, and +3.4% for E_95%_, E_50%_, and E_5%_, respectively (Figures 4A, C, E); no GTVs were modeled in the pelvic body models. Similarly, SAR_95%_, SAR_50%_, and SAR_5%_ increased +6.5%, +10.9%, and +10.7%, respectively (Supplementary Figures 7A, C, E), while the CD_95%_, CD_50%_, and CD_5%_ increased +2.5%, +6.4%, and +3.6% (Supplementary Figures 8A, C, E). The average increase for the CTVs (n = 6) in both thoracic and pelvic body models were +8.9%, +8.0%, and +3.7% for E_95%_, E_50%_, and E_5%_, respectively (Figures 4A, C, E). Interestingly, the average increase in the SAR_95%_ and SAR_50%_ were +27.0% and +17.3%, but there was an average decrease of −4.5% in the SAR_5%_ (Supplementary Figures 7A, C, E). The average CD metrics, CD_95%_, CD_50%_, and CD_5%_, increased by +16.1%, +7.8%, and +3.1%, respectively (Supplementary Figures 8A, C, E). Together, both the thoracic and pelvis models demonstrate that E, SAR and CD at the GTV/CTV can be maximized at 1 S/m. But this maximum is much lower compared to the head models.

### Whole skin optimization maximizes target coverage while minimizing skin dose

**FIGURE 5:**
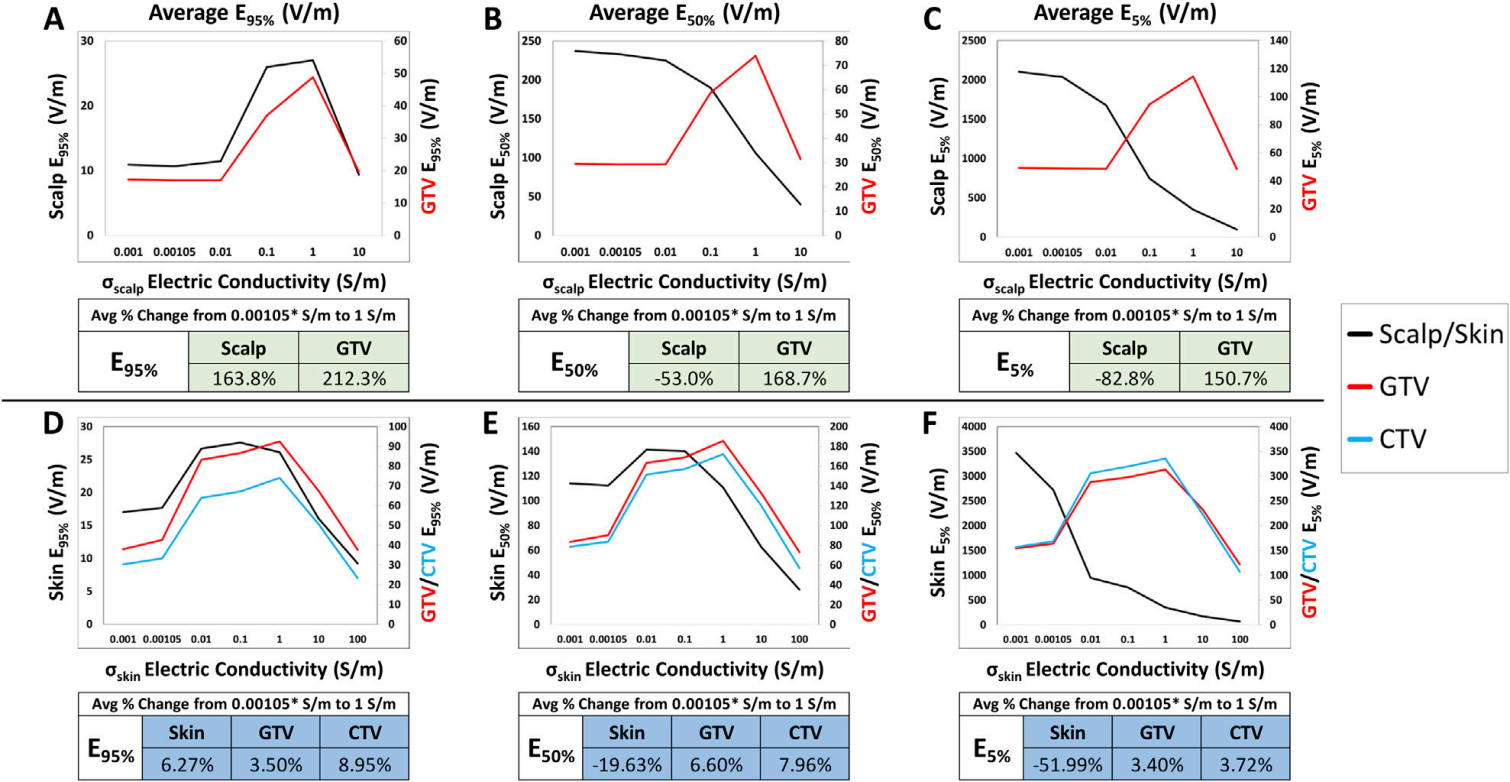
Average E_95%_, E_50%_, and E_5%_ coverage metrics between the GTV/CTV and scalp/skin with respect to varying scalp electric conductivity. Individual minimum conductivity thresholds of the whole scalp/skin and maximum coverage to the GTV/CTV are shown at the intersecting points and largest GTV to scalp/skin differences, respectively. An average of the E_95%_, E_50%_, and E_5%_ metrics of the head models are displayed with the average percent change from 0.00105 to 1.0 S/m **(A–C)** while the body models are displayed in **(D–F)**.

When comparing electric field coverage for the GTV, CTV, and scalp/skin, we observed that there are individual minimum conductivity thresholds of the whole scalp or the entire skin that can maximize TTFields coverage to the GTV while minimizing exposure on the skin (Figure 5). Importantly, maximum coverage to the GTV can be achieved with whole skin conductivity set at 1.0 S/m to minimize TTFields exposure on the skin. From the baseline conductivity of 0.00105 to 1.0 S/m, the average E_95%_ for the GTV within the head models increased approximately +212.3% while the E_95%_ of the scalp increased +163.8% (Figure 5A). More profound differences are shown in the E_50%_ and E_5%_, such that the average E_50%_ and E_5%_ for the GTV increased +168.7% and +150.7%, respectively, while the average E_50%_ and E_5%_ for the scalp decreased by −53.0% and −82.8%, respectively (Figures 5B, C). Similar trends are observed in the body models, with average E_95%_ coverage for the skin, GTV, and CTV increased +6.3%, +3.5%, and +8.9%, respectively, as the skin electric conductivity increased from 0.00105 to 1.0 S/m (Figure 5D). The average E_50%_ increased by +6.6% and +8.0% for the GTV and CTV, respectively, while the skin E_50%_ decreased by −19.6% (Figure 5E). The average E_5%_ for the GTV and CTV increased +3.4% and +3.7% for the GTV and CTV, respectively, while the skin E_5%_ decreased by −52.0% (Figure 5F). Similar trends can be seen for the SAR and current density parameters (Supplementary Figures 9, 10). In aggregate, setting the scalp or skin conductivity to 1.0 S/m maximizes target coverage while minimizing electric field exposure at the scalp or skin layers.

### Optimized TTFields distribution with respect to locally injected conductive fluids compared with whole scalp conductivity changes

**FIGURE 6:**
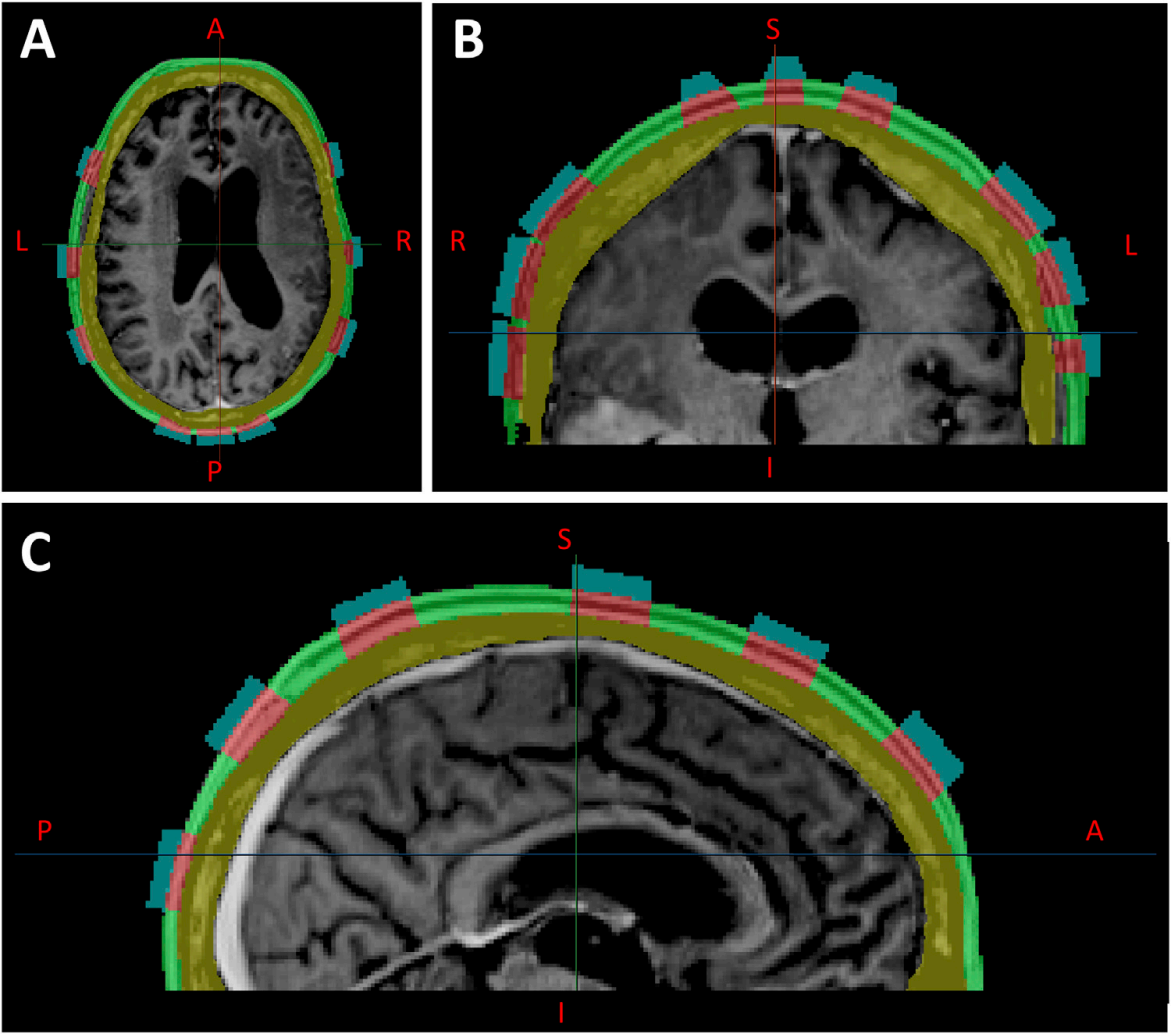
Locally injected fluids within the scalp (green layer), segmented directly below each hydrogel layer, shown in axial **(A)**, coronal **(B)**, and sagittal **(C)** planes.

**FIGURE 7:**
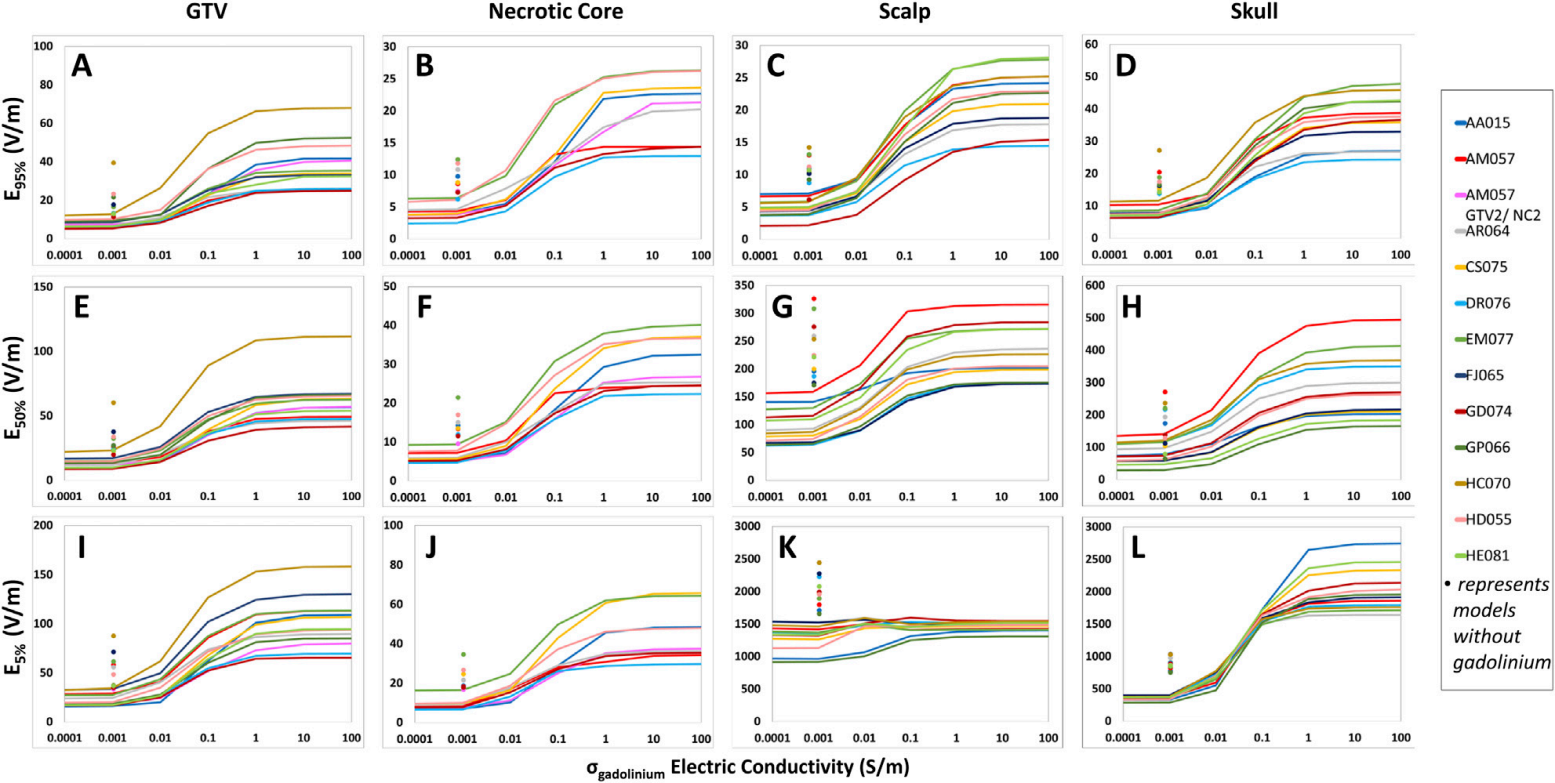
Differences in the conductive fluid conductivity saturation characteristics among GTV, necrotic core, scalp and skull of the head models according to E_95%_, E_50%_, and E_5%_ coverage metrics. For each measured structure, E_95%_ **(A–D)**, E_50%_ **(E–H)**, and E_5%_ **(I–L)** increased as the scalp electric conductivity rose from 0.1 to 1.0 S/m, but no further increase was noted beyond 1.0 S/m. Additionally, each point at 0.00105 S/m displays the electric field coverages at the baseline scalp electrical conductivity without the presence of gadolinium.

Changes in coverage metrics due to locally injected fluids within the scalp, particularly in the volume of tissue directly below each hydrogel layer were investigated and subsequently compared with coverage metrics from whole scalp conductivity changes as previously described (Figure 6). As a result, TTFields distribution was shown to saturate in the head models at approximately 1.0 S/m for both the GTV and scalp, with negligible change beyond 1.0 S/m. This saturation point was consistent across the EVH, CDVH, and SARVH 95%, median or 50%, and hotspot or 5% coverages (Figure 7; Supplementary Figures 11, 12). Notably, the E_95%_, E_50%_, and E_5%_ coverages at the GTV increased with averages of +107.3%, +89.7%, and +85.1%, respectively, when increasing the conductivity from 0.00105 to 1.0 S/m. In contrast, the E_50%_ and E_5%_ coverages at the scalp decreased at averages of −4.0% and −25.6% respectively, although the E_95%_ increased by +93.2%.

In comparison to whole scalp conductivity alteration, at the conductivity saturation point of 1.0 S/m, the average E_95%_, E_50%_, and E_5%_ of the GTV increased by +44.6%, +34.4%, and +23.9%, respectively (Table 1). At approximately 3.0 S/m, through linear interpolation, the GTV increased +23.9%, +16.0%, and +7.1% in E_95%_, E_50%_, and E_5%_. As for the scalp at 1.0 S/m, E_95%_ increased +40.3% but decreased −50.6% and −75.8% for E_50%_ and E_5%_ at 1.0 S/m, respectively. At 3.0 S/m, the scalp increased +18.5% at E_95%_ but decreased −57.6% and −79.7% for E_50%_ and E_5%_, respectively. Tables 2, 3 showed average SAR_95%_, SAR_50%_, SAR_5%_, CD_95%_, CD_50%_, and CD_5%_, respectively.

**TABLE 1:** Average E_95%_, E_50%_, and E_5%_ scalp and GTV percent change from locally injected fluids within the scalp to whole scalp conductivity change.

σ_scalp_, Electric conductivity (S/m)		1	2	3	10	1	2	3	10
		Scalp				GTV			
E_95%_	AA015	−35.2%	−41.4%	−47.5%	−89.0%	16.0%	5.7%	−4.5%	−70.9%
	AM057 GTV1	17.0%	7.5%	−2.0%	−65.5%	9.8%	1.7%	−6.4%	−62.0%
	AM057 GTV2					94.0%	79.7%	65.8%	−22.8%
	AR064	68.9%	56.2%	43.6%	−40.9%	61.6%	50.4%	39.3%	−36.0%
	CS075	39.0%	27.5%	16.1%	−59.8%	67.9%	56.9%	46.1%	−26.8%
	DR076	111.4%	95.9%	80.5%	−23.6%	33.7%	24.7%	15.8%	−45.0%
	EM077	20.6%	10.9%	1.4%	−62.7%	37.2%	27.2%	17.2%	−50.9%
	FJ065	66.0%	53.1%	40.4%	−45.0%	26.9%	17.9%	8.9%	−52.5%
	GD074	101.1%	83.0%	65.3%	−46.8%	116.3%	102.1%	88.1%	−7.0%
	GP066	9.3%	0.6%	−8.0%	−64.9%	3.8%	−4.6%	−12.8%	−68.4%
	HC070	15.5%	7.1%	−1.1%	−56.0%	−0.6%	−7.1%	−13.6%	−58.2%
	HD055	31.6%	21.5%	11.6%	−54.8%	42.5%	33.5%	24.7%	−35.1%
	HE081	8.0%	1.1%	−5.6%	−50.5%	52.9%	39.7%	26.9%	−52.3%
	AVERAGE	40.3%	29.3%	18.5%	−53.5%	44.6%	34.2%	23.9%	−44.8%
	MEDIAN	26.1%	16.2%	6.5%	−55.4%	37.2%	27.2%	17.2%	−50.9%
E_50%_	AA015	−60.2%	−63.5%	−66.7%	−89.3%	15.9%	6.1%	−3.5%	−66.7%
	AM057 GTV1	−63.7%	−66.3%	−68.9%	−87.0%	4.1%	−3.7%	−11.5%	−64.7%
	AM057 GTV2					87.0%	73.7%	60.5%	−25.2%
	AR064	−51.0%	−54.6%	−58.2%	−82.8%	63.2%	53.2%	43.3%	−23.4%
	CS075	−50.2%	−53.8%	−57.5%	−82.6%	49.8%	39.4%	29.1%	−38.3%
	DR076	−37.8%	−42.0%	−46.2%	−74.9%	25.6%	17.2%	8.9%	−47.3%
	EM077	−58.2%	−61.1%	−64.1%	−84.4%	19.9%	11.3%	2.7%	−55.1%
	FJ065	−38.7%	−43.1%	−47.5%	−77.2%	18.1%	9.8%	1.6%	−54.2%
	GD074	−62.1%	−64.9%	−67.6%	−86.8%	76.5%	65.4%	54.5%	−18.5%
	GP066	−46.8%	−50.8%	−54.8%	−82.2%	0.1%	−7.0%	−14.1%	−62.7%
	HC070	−45.0%	−48.6%	−52.1%	−76.4%	−6.1%	−12.3%	−18.4%	−60.4%
	HD055	−44.2%	−48.1%	−52.0%	−78.8%	36.9%	28.1%	19.5%	−39.2%
	HE081	−54.8%	−57.8%	−60.7%	−81.0%	31.2%	22.7%	14.3%	−42.2%
	AVERAGE	−50.6%	−54.1%	−57.6%	−81.7%	34.4%	25.1%	16.0%	−45.1%
	MEDIAN	−50.6%	−54.2%	−57.8%	−82.4%	31.2%	22.7%	14.3%	−47.3%
E_5%_	AA015	−84.4%	−85.8%	−87.2%	−96.7%	−10.5%	−18.0%	−25.4%	−73.7%
	AM057 GTV1	−74.4%	−76.5%	−78.6%	−93.3%	8.5%	0.4%	−7.7%	−62.7%
	AM057 GTV2					80.1%	67.4%	54.9%	−26.7%
	AR064	−77.3%	−79.2%	−81.1%	−94.3%	27.4%	19.5%	11.7%	−41.5%
	CS075	−76.4%	−78.3%	−80.2%	−93.8%	35.5%	26.7%	18.0%	−38.9%
	DR076	−75.4%	−77.4%	−79.3%	−92.7%	20.4%	12.7%	5.0%	−47.6%
	EM077	−73.8%	−75.9%	−78.0%	−92.7%	2.9%	−4.4%	−11.8%	−61.9%
	FJ065	−76.6%	−78.4%	−80.3%	−93.6%	11.0%	2.9%	−5.1%	−59.1%
	GD074	−77.1%	−79.0%	−80.8%	−93.8%	54.7%	45.4%	36.1%	−28.0%
	GP066	−75.9%	−77.9%	−79.9%	−93.8%	0.9%	−6.7%	−14.3%	−65.4%
	HC070	−71.4%	−73.6%	−75.8%	−91.3%	−4.9%	−11.1%	−17.3%	−59.6%
	HD055	−75.3%	−77.3%	−79.2%	−93.1%	44.8%	35.7%	26.6%	−34.4%
	HE081	−76.4%	−78.1%	−79.9%	−91.9%	25.3%	16.9%	8.6%	−47.4%
	AVERAGE	−75.8%	−77.8%	−79.7%	−93.3%	23.9%	15.5%	7.1%	−49.3%
	MEDIAN	−76.1%	−78.0%	−79.9%	−93.4%	20.4%	12.7%	5.0%	−47.6%

**TABLE 2:** Average SAR_95%_, SAR_50%_, and SAR_5%_ scalp and GTV percent change from to locally injected fluids within the scalp to whole scalp conductivity change.

σ_scalp_, Electric conductivity (S/m)		1	2	3	10	1	2	3	10
		Scalp				GTV			
SAR_95%_	AA015	20,223%	18,694%	17,167%	6,500%	40.6%	23.5%	7.1%	−92.3%
	AM057 GTV1	63,864%	63,242%	62,626%	58,466%	19.5%	7.7%	−3.9%	−80.8%
	AM057 GTV2					262.5%	223.2%	185.3%	−45.1%
	AR064	85,674%	88,823%	91,958%	113,528%	131.5%	108.9%	86.7%	−61.0%
	CS075	82,379%	81,399%	80,423%	73,729%	208.5%	176.7%	146.0%	−40.3%
	DR076	106,849%	111,693%	116,525%	150,000%	70.8%	53.7%	37.0%	−70.9%
	EM077	86,300%	86,471%	86,640%	87,759%	87.1%	67.0%	47.3%	−82.4%
	FJ065	103,251%	105,471%	107,678%	122,773%	61.3%	44.8%	28.6%	−77.7%
	GD074	74,785%	73,520%	72,261%	63,598%	376.3%	328.7%	282.6%	−3.6%
	GP066	57,541%	58,524%	59,500%	66,145%	5.3%	−6.6%	−18.3%	−94.2%
	HC070	73,632%	78,702%	83,741%	118,170%	−1.6%	−11.0%	−20.3%	−82.6%
	HD055	91,788%	94,753%	97,702%	117,878%	100.6%	81.2%	62.1%	−62.6%
	HE081	63,872%	72,942%	81,935%	142,818%	126.7%	104.3%	82.0%	−69.0%
	AVERAGE	75,847%	77,853%	78,254%	92,219%	114.5%	92.5%	43.1%	−72.8%
	MEDIAN	78,582%	80,050%	88,620%	107,753%	87.1%	67.0%	74.6%	−77.1%
SAR_50%_	AA015	16,300%	15,826%	15,355%	12,119%	33.5%	18.4%	3.7%	−88.7%
	AM057 GTV1	12,768%	13,179%	13,589%	16,424%	8.8%	−2.0%	−12.6%	−83.3%
	AM057 GTV2					252.2%	214.6%	178.3%	−43.4%
	AR064	24,521%	25,135%	25,741%	29,766%	163.8%	139.8%	116.2%	−39.3%
	CS075	23,883%	24,467%	25,045%	28,930%	133.6%	109.2%	85.5%	−61.3%
	DR076	39,338%	42,163%	44,958%	63,705%	56.7%	41.7%	27.0%	−69.6%
	EM077	16,377%	17,113%	17,844%	22,841%	43.8%	29.1%	14.7%	−78.9%
	FJ065	38,144%	39,654%	41,139%	50,928%	40.0%	26.0%	12.3%	−79.0%
	GD074	13,867%	14,179%	14,490%	16,615%	212.4%	182.2%	152.6%	−38.3%
	GP066	25,897%	26,220%	26,539%	28,678%	0.8%	−10.1%	−20.7%	−89.8%
	HC070	29,250%	32,100%	34,922%	53,896%	−14.7%	−22.8%	−30.8%	−84.5%
	HD055	30,001%	31,533%	33,049%	43,231%	109.7%	89.2%	69.1%	−62.6%
	HE081	19,588%	21,317%	23,032%	34,688%	76.2%	58.9%	41.9%	−66.9%
	AVERAGE	24,161%	25,241%	23,313%	29,979%	85.9%	67.2%	28.1%	−73.1%
	MEDIAN	24,202%	24,801%	28,180%	34,083%	56.7%	41.7%	47.0%	−70.2%
SAR_5%_	AA015	2,607%	2,438%	2,259%	1,092%	−14.2%	−24.1%	−33.7%	−92.7%
	AM057 GTV1	6,678%	6,428%	6,178%	4,459%	24.0%	10.8%	−2.1%	−86.4%
	AM057 GTV2					214.4%	179.5%	146.2%	−49.5%
	AR064	5,005%	4,805%	4,606%	3,222%	52.9%	39.0%	25.2%	−67.4%
	CS075	5,796%	5,611%	5,426%	4,151%	82.1%	64.2%	46.9%	−62.3%
	DR076	6,562%	6,469%	6,377%	5,731%	61.0%	45.2%	29.7%	−72.9%
	EM077	7,258%	7,039%	6,820%	5,297%	10.5%	−0.9%	−12.2%	−85.3%
	FJ065	5,548%	5,402%	5,256%	4,241%	20.4%	8.4%	−3.5%	−83.3%
	GD074	5,465%	5,302%	5,139%	4,010%	139.7%	118.2%	96.9%	−47.8%
	GP066	6,828%	6,565%	6,303%	4,513%	3.0%	−7.9%	−18.5%	−87.3%
	HC070	8,579%	8,513%	8,446%	7,982%	−9.0%	−17.7%	−26.3%	−83.5%
	HD055	6,406%	6,234%	6,063%	4,880%	91.8%	73.8%	56.1%	−60.4%
	HE081	5,889%	5,992%	6,094%	6,801%	43.4%	29.8%	16.3%	−73.3%
	AVERAGE	6,052%	5,900%	5,790%	4,773%	55.4%	39.9%	10.3%	−76.9%
	MEDIAN	6,148%	6,113%	5,725%	4,314%	43.4%	29.8%	16.3%	−73.3%

**TABLE 3:** Average CD_95%_, CD_50%_, and CD_5%_ scalp and GTV percent change from locally injected fluids within the scalp to whole scalp conductivity change.

σ_scalp_, Electric conductivity (S/m)		1	2	3	10	1	2	3	10
		Scalp				GTV			
CD_95%_	AA015	6,173%	6,651%	7,122%	10,243%	6.1%	−2.3%	−10.6%	−66.6%
	AM057 GTV1	11,100%	13,623%	16,119%	32,869%	10.9%	2.4%	−6.1%	−63.8%
	AM057 GTV2					76.0%	64.3%	52.8%	−22.4%
	AR064	14,238%	18,442%	22,612%	50,898%	54.1%	44.0%	33.9%	−34.6%
	CS075	13,394%	16,377%	19,333%	39,292%	62.0%	51.7%	41.6%	−25.1%
	DR076	18,694%	24,423%	30,127%	69,357%	33.5%	25.0%	16.5%	−40.7%
	EM077	11,389%	14,126%	16,809%	34,,241%	27.7%	19.2%	10.7%	−47.1%
	FJ065	15,840%	20,138%	24,400%	53,269%	25.7%	17.1%	8.5%	−49.6%
	GD074	25,393%	30,592%	35,679%	68,444%	83.8%	73.8%	63.9%	−2.5%
	GP066	10,338%	13,043%	15,704%	33,219%	0.3%	−5.9%	−12.1%	−54.8%
	HC070	10,339%	13,815%	17,258%	40,479%	1.1%	−5.4%	−11.9%	−55.5%
	HD055	13,402%	17,240%	21,033%	46,423%	47.8%	39.5%	31.2%	−26.0%
	HE081	9,776%	13,879%	17,906%	44,111%	44.5%	34.5%	24.6%	−43.1%
	AVERAGE	13,340%	16,862%	20,342%	43,570%	36.4%	27.5%	18.7%	−40.9%
	MEDIAN	12,391%	15,251%	18,619%	42,295%	33.5%	25.0%	16.5%	−43.1%
CD_50%_	AA015	3,140%	3,757%	4,373%	8,649%	−3.5%	4.8%	14.6%	213.8%
	AM057 GTV1	2,844%	3,695%	4,545%	10,464%	−5.7%	2.1%	11.4%	193.0%
	AM057 GTV2					−44.8%	−41.0%	−36.7%	27.2%
	AR064	3,911%	5,055%	6,193%	13,996%	−37.8%	−33.7%	−29.1%	32.5%
	CS075	3,989%	5,147%	6,299%	14,239%	−38.0%	−33.8%	−29.0%	37.8%
	DR076	4,973%	6,717%	8,453%	20,360%	−10.8%	−5.1%	1.3%	90.5%
	EM077	3,299%	4,338%	5,374%	12,547%	−15.3%	−8.8%	−1.3%	124.5%
	FJ065	4,907%	6,460%	8,006%	18,597%	−17.4%	−11.1%	−3.8%	115.9%
	GD074	2,993%	3,856%	4,715%	10,642%	−37.7%	−33.6%	−29.0%	32.4%
	GP066	4,251%	5,408%	6,561%	14,495%	−0.6%	6.8%	15.3%	152.9%
	HC070	4,397%	6,092%	7,778%	19,350%	8.5%	15.7%	23.9%	137.2%
	HD055	4,499%	5,946%	7,386%	17,301%	−36.4%	−32.3%	−27.7%	36.1%
	HE081	3,606%	4,950%	6,287%	15,486%	−19.5%	−14.2%	−8.2%	76.6%
	AVERAGE	3,901%	5,118%	6,331%	14,677%	−19.9%	−14.2%	−7.6%	97.7%
	MEDIAN	3,950%	5,101%	6,293%	14,367%	−17.4%	−11.1%	−3.8%	90.5%
CD_5%_	AA015	1,260%	1,425%	1,588%	2,716%	5.5%	15.4%	27.1%	287.7%
	AM057 GTV1	2,057%	2,423%	2,789%	5,330%	−12.4%	−5.7%	2.1%	127.8%
	AM057 GTV2					−45.6%	−41.5%	−36.9%	27.1%
	AR064	1,835%	2,155%	2,473%	4,696%	−19.0%	−14.7%	−10.0%	44.9%
	CS075	1,972%	2,318%	2,664%	5,069%	−25.6%	−20.8%	−15.3%	56.1%
	DR076	2,061%	2,630%	3,198%	7,160%	−21.6%	−15.9%	−9.4%	96.1%
	EM077	2,126%	2,604%	3,081%	6,408%	−5.5%	1.0%	8.5%	117.6%
	FJ065	1,935%	2,303%	2,671%	5,237%	−5.2%	1.5%	9.2%	126.5%
	GD074	1,904%	2,254%	2,603%	5,030%	−33.3%	−29.8%	−25.8%	21.3%
	GP066	2,036%	2,412%	2,788%	5,402%	−1.9%	6.8%	17.1%	217.6%
	HC070	2,326%	2,950%	3,573%	7,916%	6.6%	13.7%	21.7%	134.8%
	HD055	2,041%	2,498%	2,954%	6,123%	−26.1%	−21.6%	−16.6%	47.7%
	HE081	1,909%	2,490%	3,070%	7,095%	−15.2%	−10.2%	−4.6%	65.8%
	AVERAGE	1,955%	2,372%	2,788%	5,682%	−15.3%	−9.4%	−2.5%	105.5%
	MEDIAN	2,004%	2,418%	2,788%	5,366%	−15.2%	−10.2%	−4.6%	96.1%

## Discussion

We found that the hydrogel-scalp or hydrogel-skin interface is critical for the penetration of TTFields into the intracranial space or other body cavities, respectively. Kirson et al. found that higher TTFields intensity correlated with increased percent of tumor cell kill in experimental cell culture models (Kirson et al., 2007). To optimize delivery of TTFields for the treatment of cancer, the penetrating dose at the tumor volume(s) in the brain or torso should be maximized while the hotspots on the scalp or skin surface minimized. The conductivity of the hydrogel as well as the skin or scalp itself is speculated to alter TTFields coverage at both GTV and/or CTV as well as the hotspot on the scalp or skin. This may occur under the conditions of high ambient temperature and increased humidity. Indeed, as we increased the conductivity of hydrogel or volume of scalp directly below each hydrogel layer, TTFields intensity at the GTV or CTV reached a maximum beyond which no further increase occurred, suggesting that the scalp or other skin surface may function as a variable conductor to mitigate these effects. Interestingly, increasing the conductivity of the entire scalp or skin resulted in a peak electric-field intensity characteristics within the GTV, with a corresponding decrease in field intensity at the scalp or skin. Therefore, optimal delivery is possible by altering the conductivity of the entire scalp or skin.

The observed saturation characteristics is likely due to a relationship between electric conductivity and the response of charge carriers under the influence of time-dependent alternating electric fields. This relationship is determined by the concentration and allowable motion of charges that determine a material’s electric conductivity according to field intensity and the quantity of electric charges within an enclosed space according to Gauss’ Law. As the conductivity of the hydrogel approaches large values, its electrical response behaves similar to that of metals, where the charge carrier density can then be calculated according to the Drude model (Dressel and Scheffler, 2006). Charge carrier density is typically constant for a particular material in specific states of matter and, therefore, can only conduct a certain amount of electric charge. As we increase the electric conductivity of hydrogel, the charge carrier density increases. However, since the electric conductivity of the anatomical structures throughout the rest of the head models remains constant in this study, the intrinsic charge carrier densities are also unchanged. Therefore, increasing the conductivity of hydrogel results in saturation of electric field, SAR and CD intensities within the head models. A similar explanation is also applicable to the body models.

The saturation point for electric fields coverage as a function of hydrogel conductivity is twice as high for thorax and pelvis models compared to the head models (Wenger et al., 2015). This may be due to the higher output from the NovoTTF-100L device for the thorax and pelvis (https://www.accessdata.fda.gov/cdrh_docs/pdf18/H180002C.pdf) compared to the NovoTTF-100A or NovoTTF-200A for the head. Specifically, the maximum intensity for NovoTTF-100L is 1,414 mA while the maximum for NovoTTF-100A or NovoTTF-200A is 900 mA, or a ratio of 1.6. In addition, each NovoTTF-100L array includes up to 20 ceramic electrodes arranged in a 4 × 4 rectangular configuration with 2 additional electrodes at either side of the long axis (https://www.accessdata.fda.gov/cdrh_docs/pdf18/H180002D.pdf), compared to the 9 electrodes in a rectangular 3 × 3 configuration for NovoTTF-100A or NovoTTF-200A (https://www.accessdata.fda.gov/cdrh_docs/pdf10/P100034c.pdf). Furthermore, the girth of body cavities is larger than the head and thus requiring higher injected current for deeper penetration. Therefore, the combination of higher electric output, greater number of electrodes, and increased required current injection could contribute to the higher saturation point for hydrogel conductivity in the thoracic and pelvic models.

One of the limitations of our study is that we did not discern between the basal and the dermal skin layers, both of which have unique physical and physiological properties that may account for differences in electric field interactions. Skin dosimetry within radiation oncology has been a continued challenge from the perspective of determining toxicity and their associated effects such as erythema, desquamation, and necrosis (Lee et al., 2022; Hoppe et al., 2008). In fact, the International Commission on Radiological Protection (ICRP) recommends evaluating the skin dose at both the basal and dermal layers separately at different depths (ICRP, 1977; ICRP, 1982; ICRP, 1992). Although ionizing radiotherapy and TTFields interact with biological tissue differently, treatments from both modalities have been reported to generate varying amounts of erythema in different patients (Lok et al., 2015; Lukas et al., 2017; Butcher and Williamson, 2021). These differences and varying effects would warrant further studies dedicated to identifying the specific interaction of TTFields at different layers of the skin. From a biologic perspective, scalp and skin could respond by hypervascularization to counteract increasing doses of applied TTFields. Hyper-vascularized scalp or skin could shunt the electric fields away from the depth of skin beneath the electrodes, causing the fields to scatter tangentially away from the point of contact rather than penetrating perpendicularly into the scalp or skin. In our prior report, scalp erythema was noted in a lateral or tangential fashion rather than localized just underneath the electrodes (Lok et al., 2015). It is probable to employ a simple approach that may be readily available, which is by applying local topicals to increase moisturization, especially using products with higher concentrations of glycerin, within the stratum corneum of the skin (Verdier-Sévrain and Bonté, 2007; Crowther et al., 2008; Loden, 2012). That is, by increasing moisturization within the epidermis, wetter skin has demonstrated increased electric conductivity and thus allow increased penetration of TTFields while minimizing charge retention within the epidermis (Wickett and Damjanovic, 2017; Li et al., 2001; Zhang et al., 2005).

There are differences in the PQMs for electric field, SAR and CD among individual head, thorax, and pelvis models. This type of individual variability indicates that TTFields coverage at the GTV or CTV are modulated by other parameters, including the geometry or location of the tumor, presence or absence of a necrotic core, association with cerebral edema, and proximity to a source of fluid such as the ventricles or bladder. First, tumors possessing asymmetry and angulated geometry have higher electric field values compared to those with symmetry and less angulation (Lok et al., 2017). This is most likely from charges accumulating at the sharp corners resulting in a higher electric force in certain regions of the tumor and therefore achieving a greater anti-tumor effect. Second, the presence of a necrotic core concentrates the electric fields to this intra-tumoral fluid-filled space and thus enabling the GTV to accumulate a greater amount of TTFields (Lok et al., 2017). Lastly, when the tumor is adjacent to ventricles or bladder, higher charges within these fluid-filled cavities may concentrate TTFields to the respective GTV or CTV (Lok et al., 2017; Lok et al., 2021). Consequently, tumor-associated or organ-specific characteristics modulate the distribution of TTFields within the intracranial, intrathoracic and intrapelvic sites.

There are potential variables that may alter TTFields distribution in the real-world patient population. Scalp thickness may be different among patients and scalp health, such as dandruff, velocity of hair growth, and diseases such as psoriasis and dermatitis, may alter penetration of TTFields from the surface. Tissue anisotropy may be another factor. However, the skull is a more significant attenuator of TTFields than the scalp. We believe that our approach in individualized modeling coupled with sensitivity analysis helps to mitigate these variables. Furthermore, there is a tradeoff between accuracy in individualized patient models and efficiency in modeling larger samples using an idealized brain or body segment (Ballo et al., 2019). We believe that personalized treatment or personalized TTFields planning is the best strategy to identify individual patient variabilities, and this will lead to a more accurate result. Our series that included 12 glioblastoma, 3 lung carcinoma and 2 ovarian carcinoma patients is still one of the largest in patient-specific models for the analysis of TTFields.

## Conclusion

TTFields delivery for the treatment of cancer can be modulated by the conductivity of the hydrogel and skin/scalp at the transducer-scalp or transducer-skin interface. Optimizing this aspect of TTFields delivery may increase tumor control while minimizing toxicity at the surface. Electrically conductive topical applications to the epidermis may reduce scalp or skin irritations while simultaneously maximizing delivery of TTFields to the tumor.

## Data Availability

The raw data supporting the conclusions of this article will be made available by the authors, without undue reservation.
