# Supplementary figures and images for: Hydrogel and scalp/skin conductivities impact dose from tumor treating fields
Source: Front Bioeng Biotechnol. 2025 Feb 24;13:1484317. doi: 10.3389/fbioe.2025.1484317 (PMC11891368; doi:10.3389/fbioe.2025.1484317)

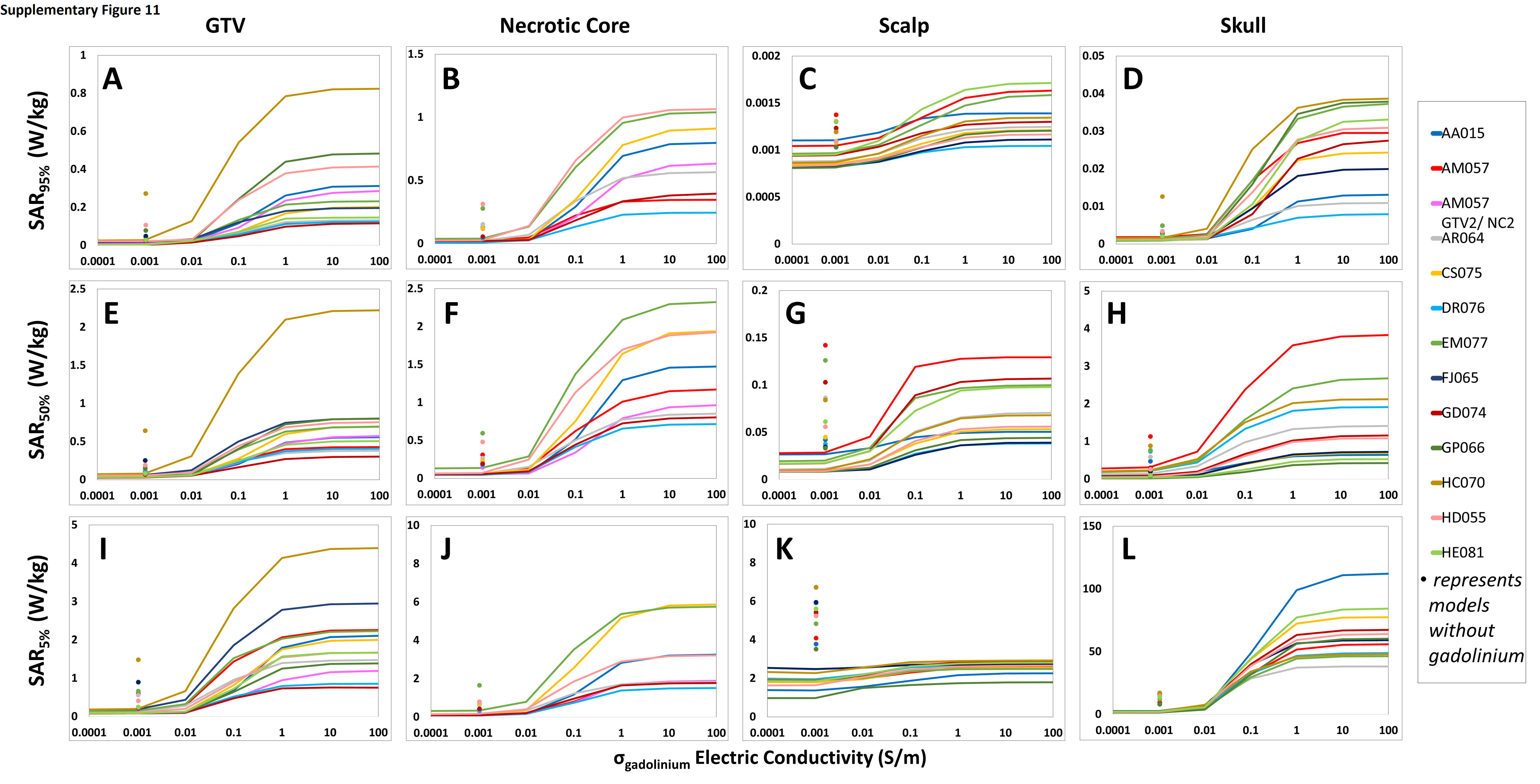

Supplement: Supplementary file 1 [file Image11.png]

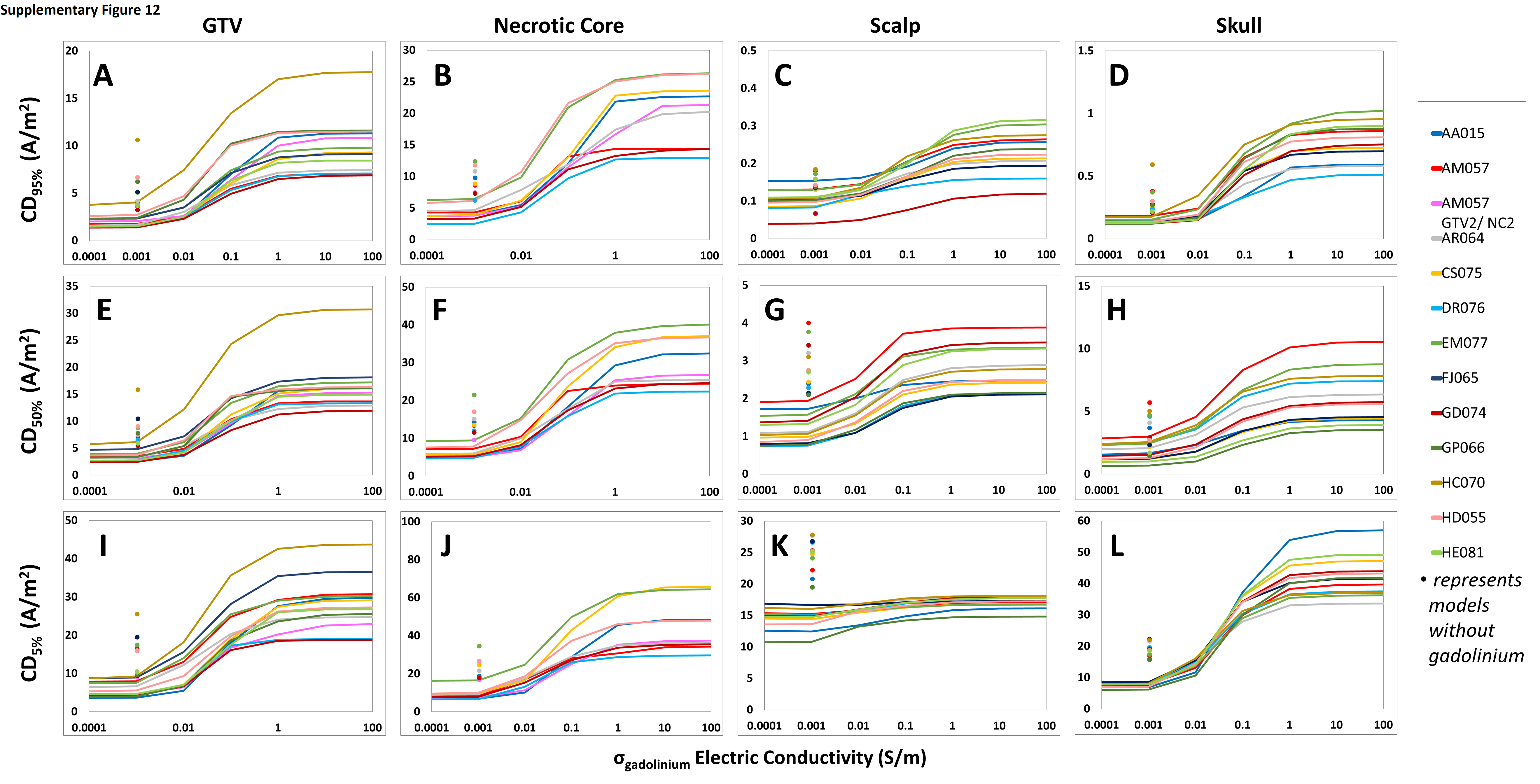

Supplement: Supplementary file 3 [file Image12.png]

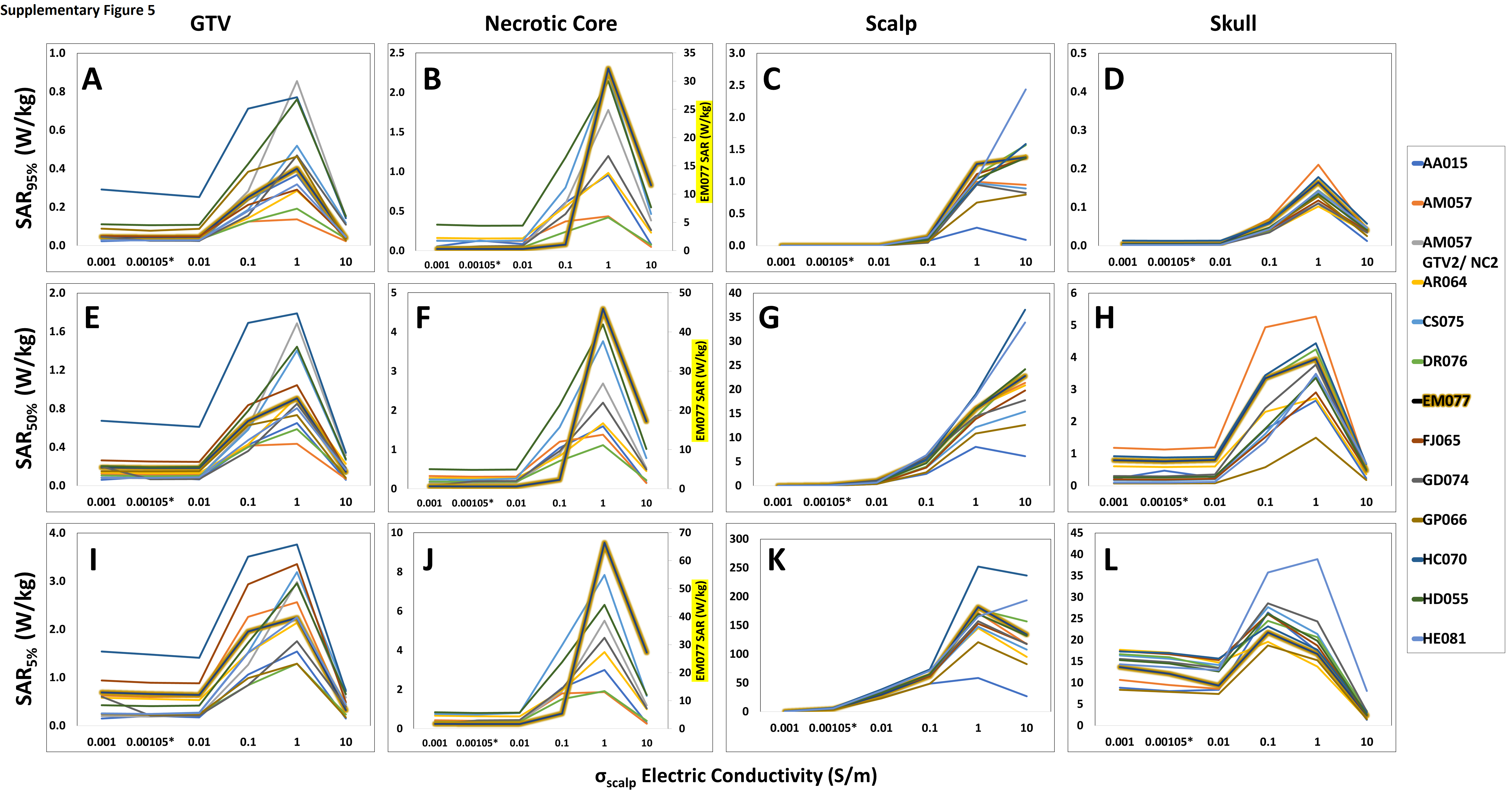

Supplement: Supplementary file 4 [file Image5.png]

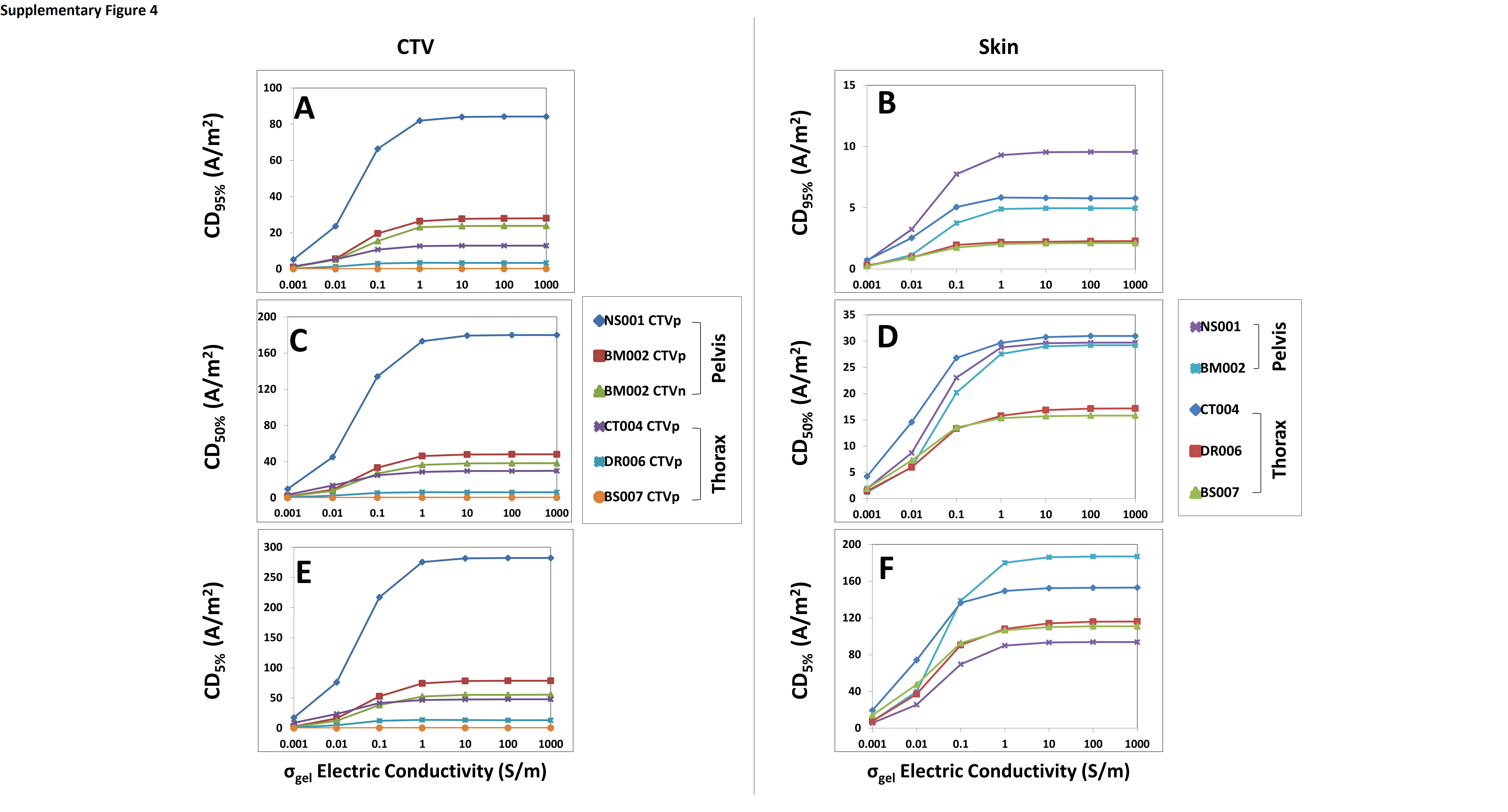

Supplement: Supplementary file 5 [file Image4.png]

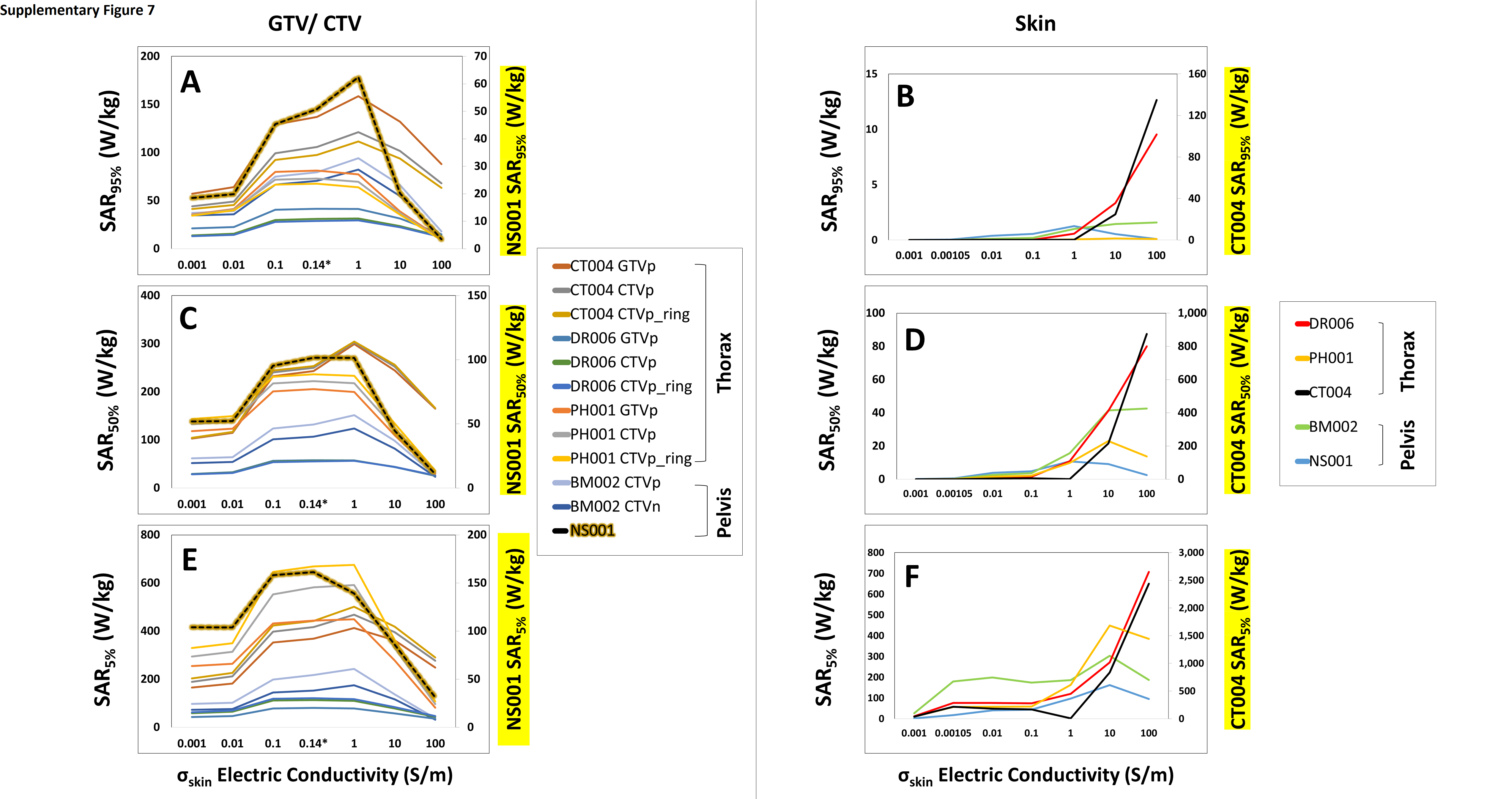

Supplement: Supplementary file 6 [file Image7.png]

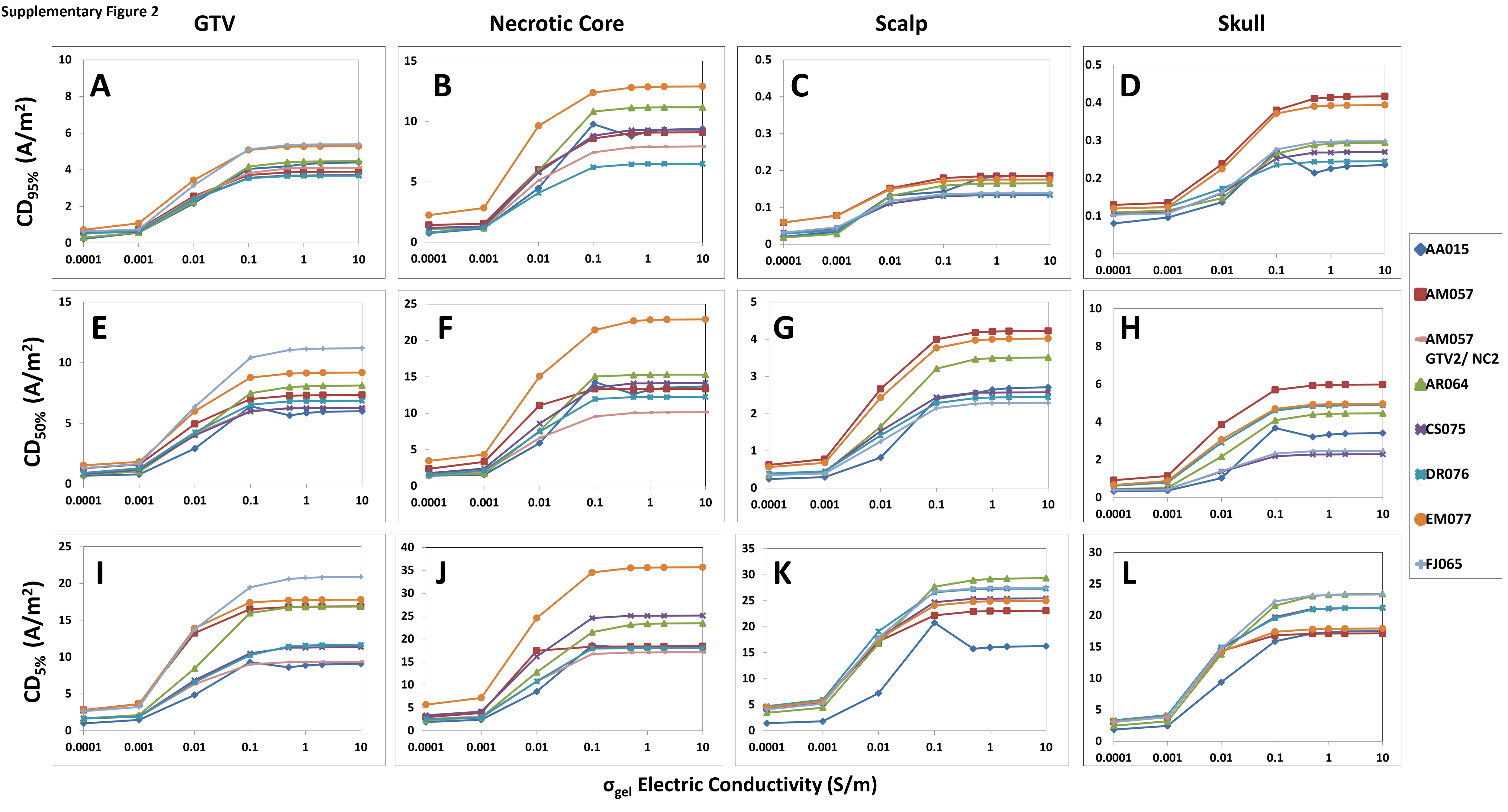

Supplement: Supplementary file 7 [file Image2.png]

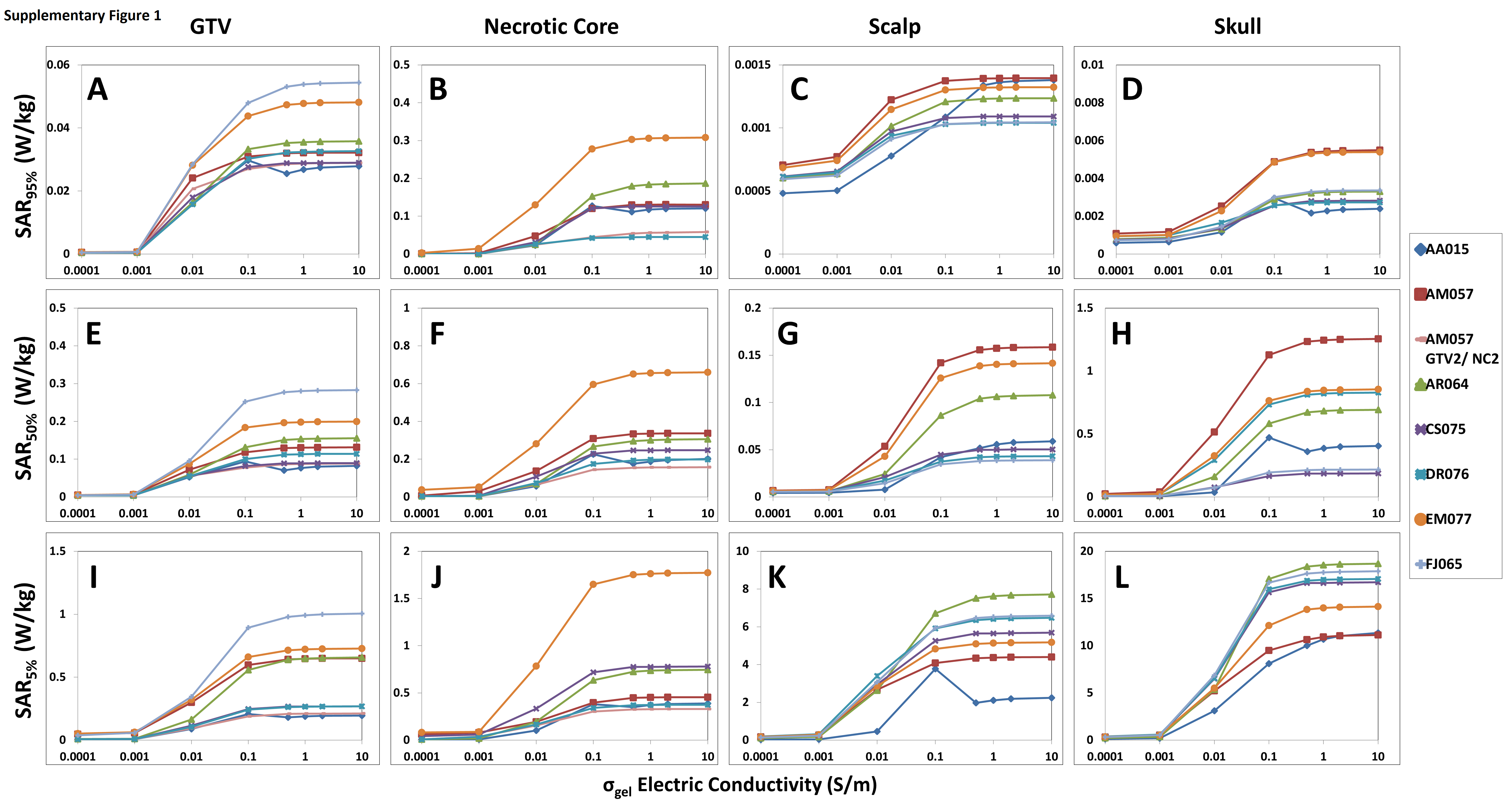

Supplement: Supplementary file 8 [file Image1.png]

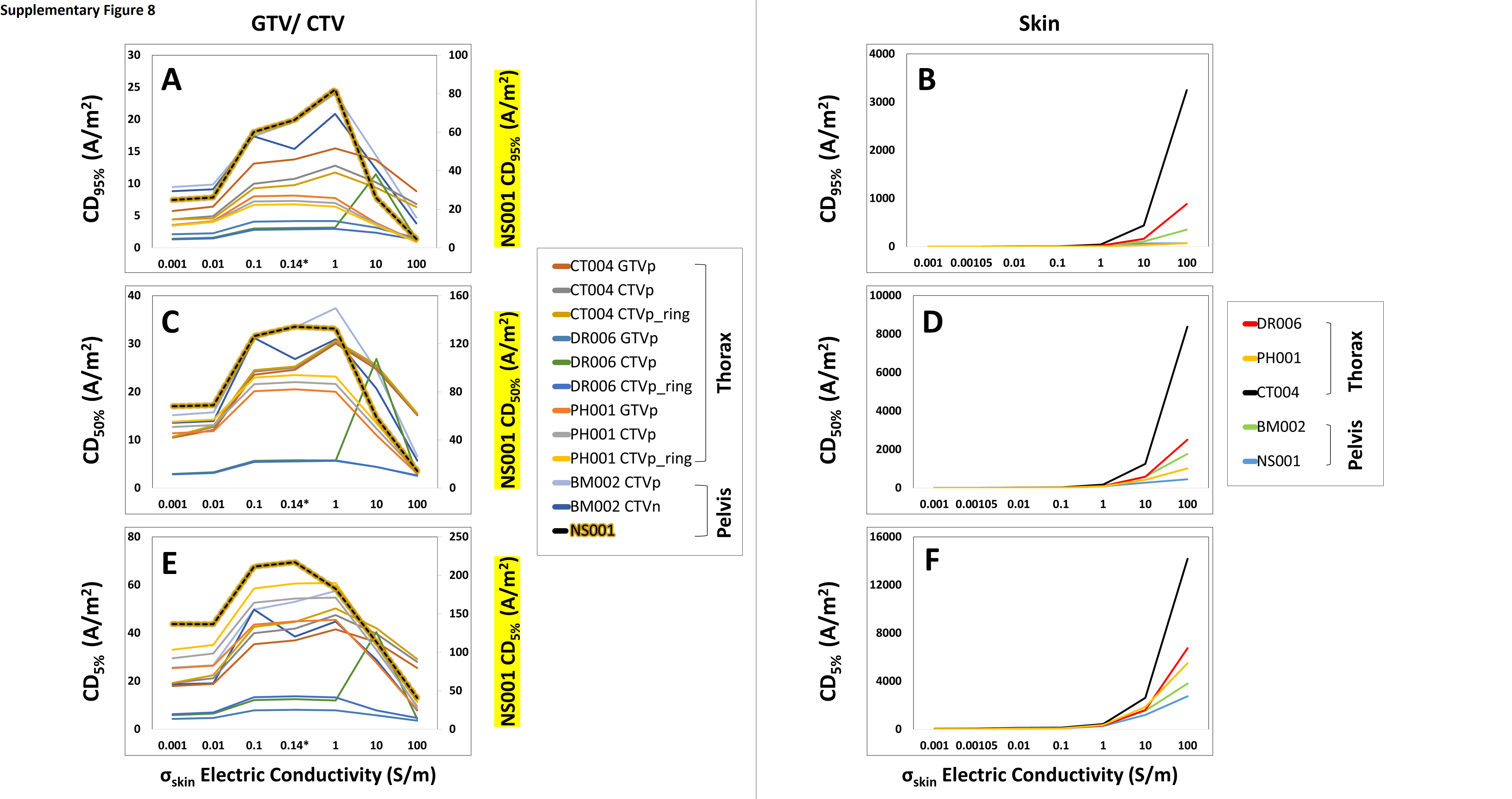

Supplement: Supplementary file 9 [file Image8.png]

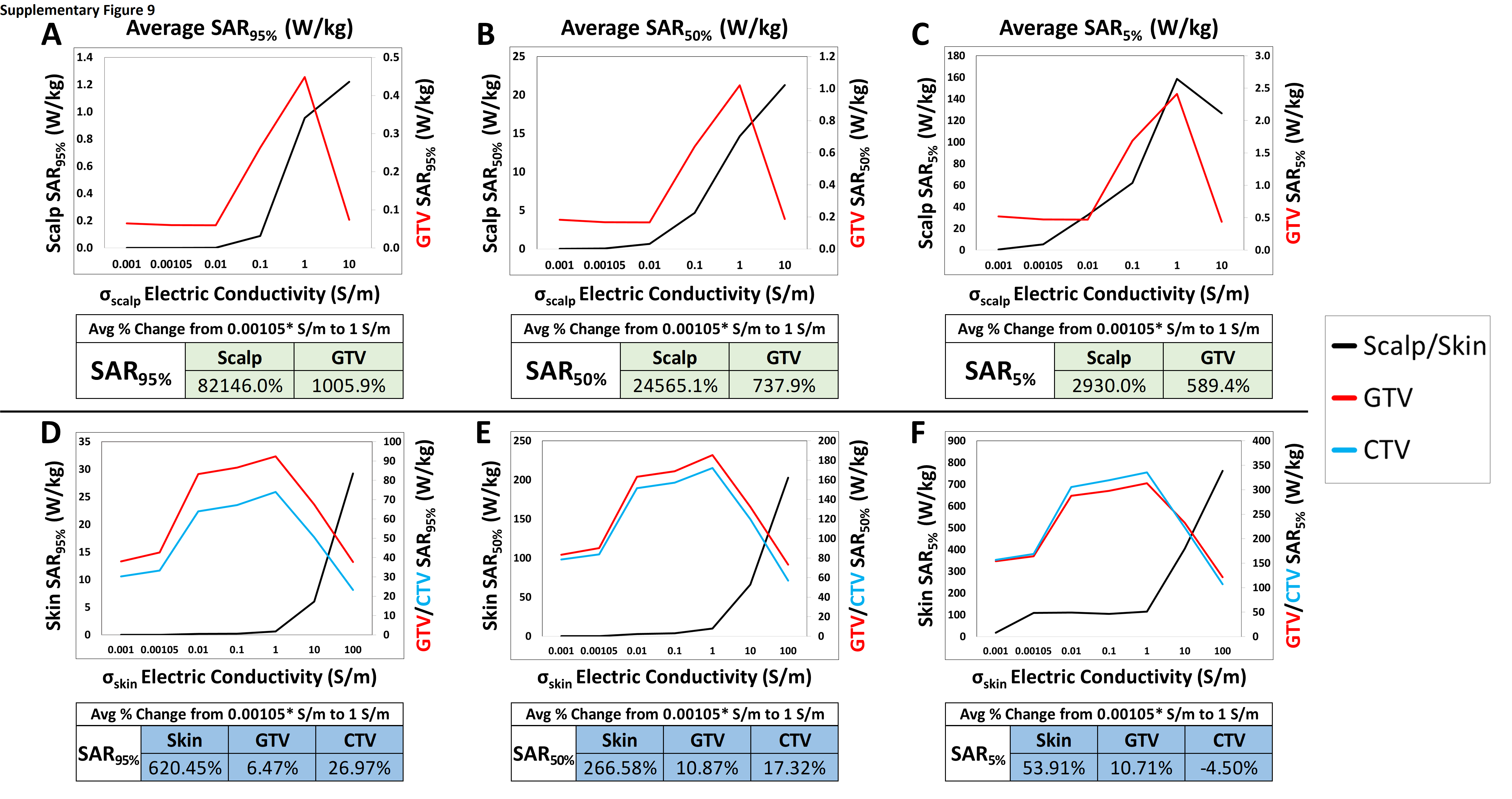

Supplement: Supplementary file 10 [file Image9.png]

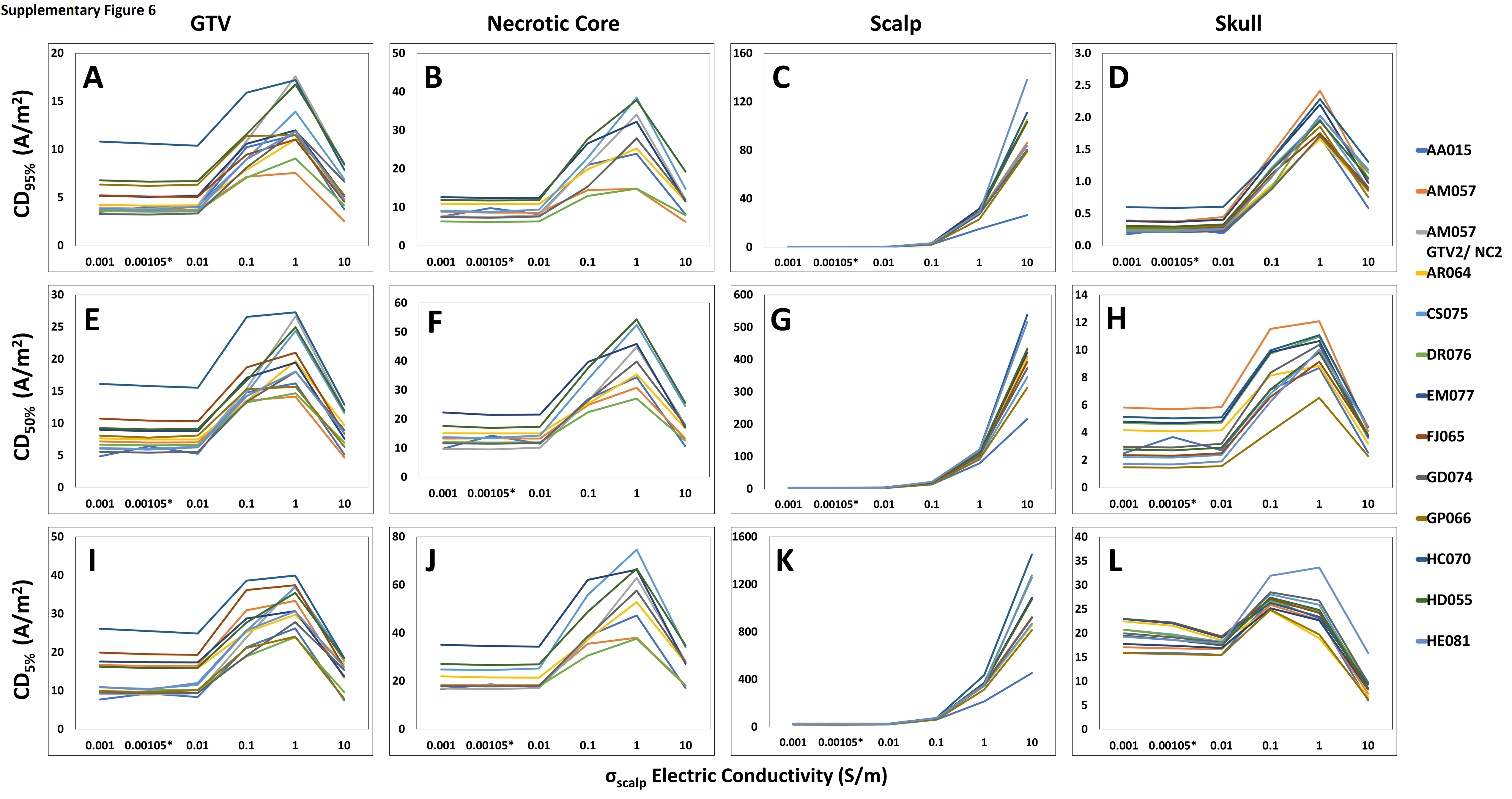

Supplement: Supplementary file 11 [file Image6.png]

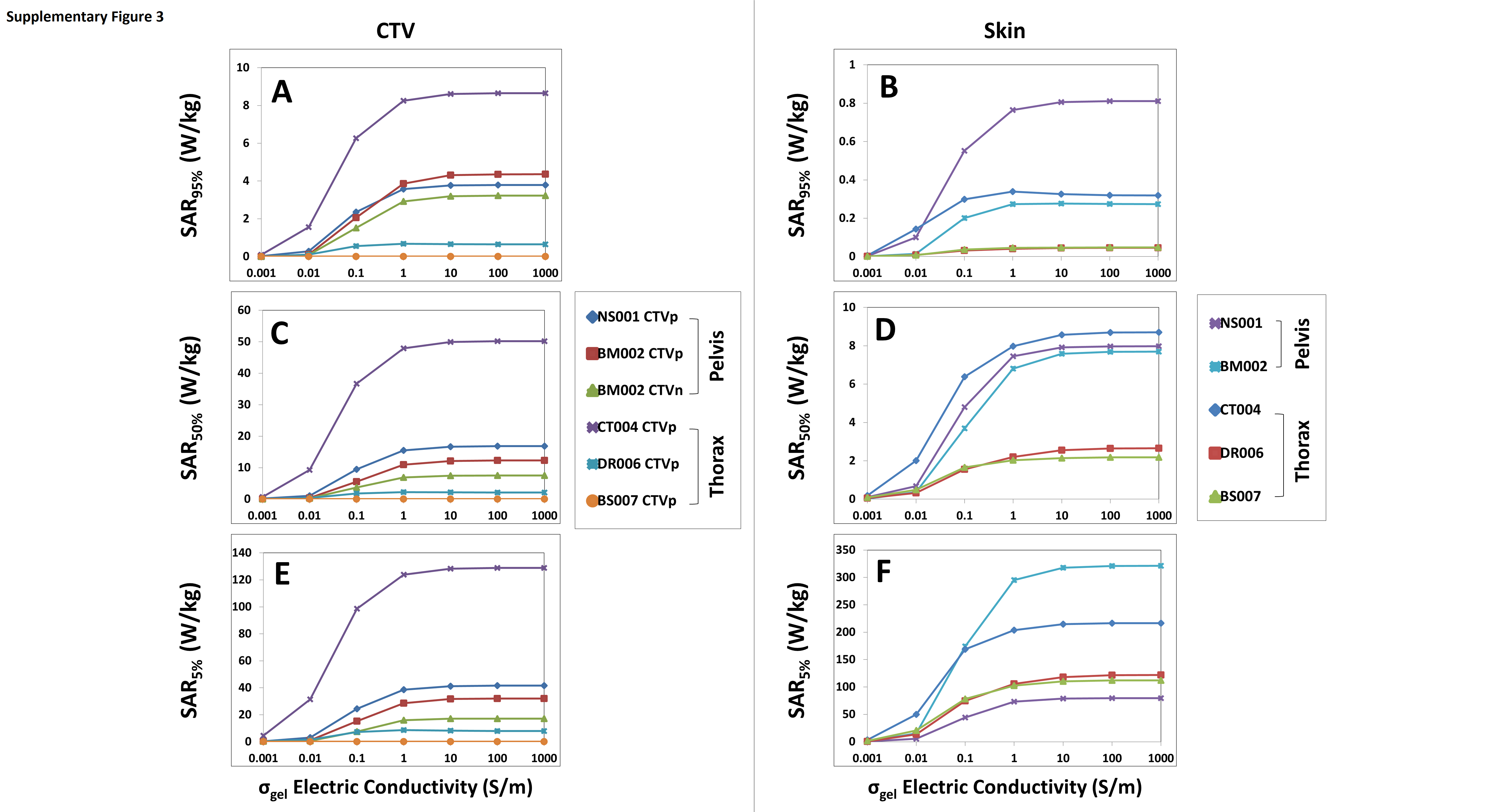

Supplement: Supplementary file 12 [file Image3.png]

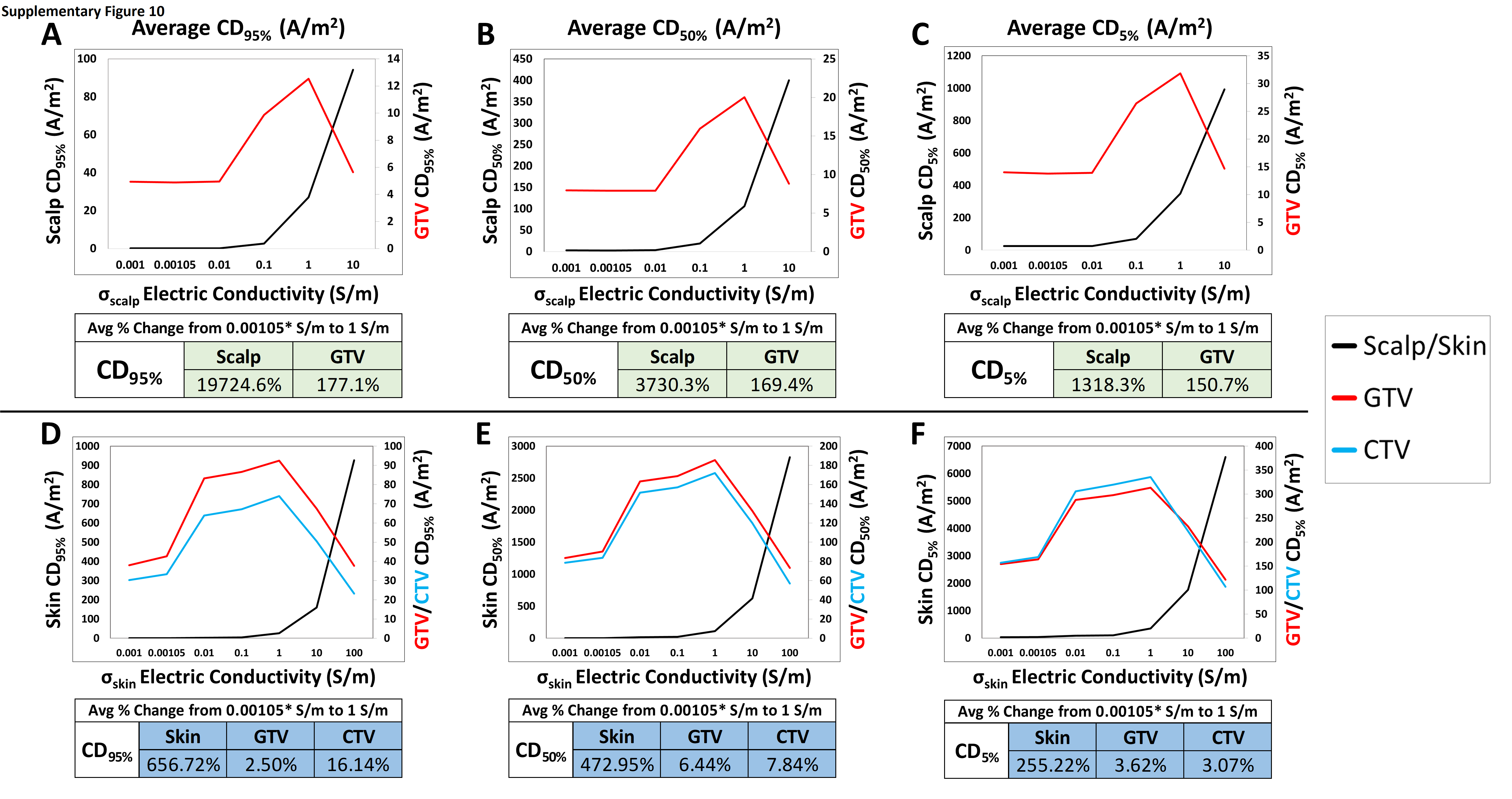

Supplement: Supplementary file 13 [file Image10.png]
