# Supplementary material for: Hydrogel and scalp/skin conductivities impact dose from tumor treating fields
Source: Front Bioeng Biotechnol. 2025 Feb 24;13:1484317. doi: 10.3389/fbioe.2025.1484317 (PMC11891368; doi:10.3389/fbioe.2025.1484317)
Supplement: Supplementary file 2 [file Supplementaryfile1.docx]

**Supplementary Figure Legends**

Supplementary Figure 1: SAR_95%_, SAR_50%_, and SAR_5%_ coverage metrics of GTV, necrotic core, scalp, and skull with respect to increasing hydrogel electric conductivity. When increasing hydrogel electric conductivity from 0.0001 to 10.0 S/m, SAR_95%_ (A-D), SAR_50%_ (E-H), and SAR_5%_ (I-L) metrics rose and saturated at 0.5 S/m. No significant increase was observed after the saturation point.

Supplementary Figure 2: CD_95%_, CD_50%_, and CD_5%_ coverage metrics of the GTV, necrotic core, scalp, and skull with respect to increasing hydrogel electric conductivity. When increasing hydrogel electric conductivity from 0.0001 to 10.0 S/m, CD_95%_ (A-D), CD_50%_ (E-H), and CD_5%_ (I-L) metrics rose until the saturation points of 0.5 S/m. No significant increase was observed after the saturation point.

Supplementary Figure 3: Difference in SAR_95%_, SAR_50%_, and SAR_5%_ coverage metrics between the CTV and skin of the body models with respect to varying hydrogel conductivity. As the hydrogel electric conductivity increased from 0.001 to 1000.0 S/m, SAR_95%_ (A-B), SAR_50%_ (C-D), and SAR_5%_ (E-F) metrics rose and reached saturation points of 1.0 S/m. No significant increase was noted after the saturation point.

Supplementary Figure 4: CD_95%_, CD_50%_, and CD_5%_ coverage metrics between the CTV and skin of the body models with respect to altering hydrogel conductivity. When increasing hydrogel electric conductivity from 0.001 to 1000.0 S/m, CD_95%_ (A-B), CD_50%_ (C-D), and CD_5%_ (E-F) metrics rose and arrived at saturation points of 1.0 S/m. No significant increase was noted after 1.0 S/m.

Supplementary Figure 5: Differences in SAR_95%_, SAR_50%_, and SAR_5%_ coverage metrics among GTV, necrotic core, scalp and skull of the head models with respect to varying skin conductivity. For the GTV, necrotic core, and skull, the SAR_95%_ (A, B, D), SAR_50%_ (E, F, H), and SAR_5%_ (I, J, L) metrics increased as the scalp conductivity increased from 0.001 to 1.0 S/m. However, a decrease was noted beyond 1.0 S/m. Similarly, the SAR_5%_ (K) for the scalp followed suit, whereas the SAR_95%_ (C) and SAR_50%_ (G) metrics had an overall increase when the scalp conductivity rose from 0.001 to 10.0 S/m. It is also notable that the necrotic core from model EM077 had distinct SAR_95%_, SAR_50%_, and SAR_5%_ coverages and its values are displayed on the secondary axis.

Supplementary Figure 6: Differences in CD_95%_, CD_50%_, and CD_5%_ coverage metrics among GTV, necrotic core, scalp and skull of the head models with respect to various skin conductivity. For the GTV, necrotic core, and skull, the CD_95%_ (A, B, D), CD_50%_ (E, F, H), and CD_5%_ (I, J, L) metrics increased as the scalp conductivity rose from 0.001 to 1.0 S/m. However, a decrease was noted beyond 1.0 S/m. Alternatively, the CD_95%_, CD_50%_, and CD_5%_ (C, G, K) for the scalp had an overall increase when the scalp conductivity increased from 0.001 to 10.0 S/m.

Supplementary Figure 7: SAR_95%_, SAR_50%_, and SAR_5%_ coverage metrics between the GTV/CTV and skin of the body models with respect to increasing skin conductivity. As the skin conductivity increased from 0.001 to 1 S/m, the GTV/CTV SAR_95%_, SAR_50%_, and SAR_5%_ coverage metrics (A, C, E) rose until 1.0 S/m and then decreased thereafter. In contrast, the skin SAR_95%_, SAR_50%_, and SAR_5%_ were shown to increase with respect to skin conductivity (B, D, F). It is also notable that the CTV from model NS001 and the skin from model CT004 had distinct SAR_95%_, SAR_50%_, and SAR_5%_ coverages, of which its values are displayed on the secondary axis.

Supplementary Figure 8: CD_95%_, CD_50%_, and CD_5%_ coverage metrics between the GTV/CTV and skin of the body models with respect to increasing skin conductivity. As the skin conductivity increased from 0.001 to 1 S/m, the GTV/CTV CD_95%_, CD_50%_, and CD_5%_ coverage metrics (A, C, E) rose until 1.0 S/m and then decreased after 1.0 S/m. In contrast, the skin CD_95%_, CD_50%_, and CD_5%_ were shown to increase with respect to skin conductivity (B, D, F). It is also notable that the CTV from model NS001 had distinct CD_95%_, CD_50%_, and CD_5%_ coverages, of which its values are displayed on the secondary axis.

Supplementary Figure 9: Average SAR_95%_, SAR_50%_, and SAR_5%_ coverage metrics between the GTV/CTV and scalp/skin with respect to varying scalp electric conductivity. Individual minimum conductivity thresholds of the whole scalp/skin and maximum coverage to the GTV/ CTV are shown at the intersecting points and largest GTV to scalp/skin differences, respectively. An average of the SAR_95%_, SAR_50%_, and SAR_5%_ metrics of the head models are displayed with the average percent change from 0.00105 to 1.0 S/m (A-C) while the body models are displayed in Figures D-F.

Supplementary Figure 10: Average CD_95%_, CD_50%_, and CD_5%_ coverage metrics between the GTV/CTV and scalp/skin with respect to varying scalp electric conductivity. Individual minimum conductivity thresholds of the whole scalp/skin and maximum coverage to the GTV/CTV are shown at the intersecting points and largest GTV to scalp/skin differences, respectively. An average of the CD_95%_, CD_50%_, and CD_5%_ metrics of the head models are displayed with the average percent change from 0.00105 to 1.0 S/m (A-C) while the body models are displayed in Figures D-F.

Supplementary Figure 11: Differences in the conductive fluid conductivity saturation characteristics among GTV, necrotic core, scalp and skull of the head models according to SAR_95%_, SAR_50%_, and SAR_5%_ coverage metrics. For each measured structure, SAR_95%_ (A-D), SAR_50%_ (E-H), and SAR_5%_ (I-L) increased as the scalp electric conductivity rose from 0.1 to 1.0 S/m. However, no further increase was noted beyond 1.0 S/m. In addition, each point at 0.00105 S/m displays the electric field coverages at the baseline scalp electrical conductivity without the presence of gadolinium.

Supplementary Figure 12: Differences in the conductive fluid conductivity saturation characteristics among GTV, necrotic core, scalp and skull of the head models according to CD_95%_, CD_50%_, and CD_5%_ coverage metrics. For each measured structure, CD_95%_ (A-D), CD_50%_ (E-H), and CD_5%_ (I-L) increased as the scalp electric conductivity rose from 0.1 to 1.0 S/m, though no further increase was noted beyond 1.0 S/m. Furthermore, each point at 0.00105 S/m displays the electric field coverages at the baseline scalp electrical conductivity without the presence of gadolinium.
